# Transcriptomic Analysis of Shiga-Toxigenic Bacteriophage Carriage Reveals a Profound Regulatory Effect on Acid Resistance in Escherichia coli

**DOI:** 10.1128/AEM.02034-15

**Published:** 2015-10-30

**Authors:** Marta Veses-Garcia, Xuan Liu, Daniel J. Rigden, John G. Kenny, Alan J. McCarthy, Heather E. Allison

**Affiliations:** Institute of Integrative Biology, University of Liverpool, Liverpool, United Kingdom

## Abstract

Shiga-toxigenic bacteriophages are converting lambdoid phages that impart the ability to produce Shiga toxin to their hosts. Little is known about the function of most of the genes carried by these phages or the impact that lysogeny has on the Escherichia coli host. Here we use next-generation sequencing to compare the transcriptomes of E. coli strains infected with an Stx phage, before and after triggering of the bacterial SOS response that initiates the lytic cycle of the phage. We were able to discriminate between bacteriophage genes expressed in the lysogenic and lytic cycles, and we describe transcriptional changes that occur in the bacterial host as a consequence of Stx phage carriage. Having identified upregulation of the glutamic acid decarboxylase (GAD) operon, confirmed by reverse transcription-quantitative PCR (RT-qPCR), we used phenotypic assays to establish the ability of the Stx prophage to confer a greater acid resistance phenotype on the E. coli host. Known phage regulators were overexpressed in E. coli, and the acid resistance of the recombinant strains was tested. The phage-encoded transcriptional regulator CII was identified as the controller of the acid response in the lysogen. Infection of an E. coli O157 strain, from which integrated Stx prophages were previously removed, showed increased acid resistance following infection with a nontoxigenic phage, ϕ24_B_. In addition to demonstrating this link between Stx phage carriage and E. coli acid resistance, with its implications for survival postingestion, the data set provides a number of other potential insights into the impact of lambdoid phage carriage on the biology of E. coli.

## INTRODUCTION

Bacteriophages have become widely recognized as important drivers of bacterial diversification and evolution (1–4). They play important roles in the adaptation of established pathogens to new metazoan hosts as well as the general emergence of new pathogens (5–7); bacteriophages can transform their commensal bacterial host to a pathogen or simply add to the virulence of pathogenic bacterial hosts (7–9), a process termed “lysogenic conversion.” In the particular case of Shiga-toxigenic Escherichia coli (STEC), especially the subset of enterohemorrhagic E. coli (EHEC), infection with Shiga toxin-encoding bacteriophages (Stx phages) was the key event leading to the emergence of these pathogens as a major health concern following their first association with an outbreak of foodborne disease in 1982 (10–12). EHEC is able to colonize the intestinal tract with great efficiency; the resulting gastrointestinal infection is potentially fatal due to the production and release of Shiga toxin, leading to hemorrhagic colitis and, in some cases, hemolytic-uremic syndrome (HUS) (12–14).

Stx phages are lambdoid phages because they possess genomes that have an organization similar to that of bacteriophage lambda (λ) (7). A recent genomic comparison of 11 Stx phages and λ revealed a high degree of mosaicism in this group, and all but one, ϕP27, had a significantly larger genome than that of λ (15). The function of this extra genomic material has yet to be determined. Between 40 and 60% of the genes carried by these 11 sequenced Stx phages are of unknown function, including genes that are highly conserved across this group (15). While Shiga toxin itself has been extensively studied (16–18) and putatively assigned the biological role of protecting a STEC population from predation by grazing protozoa (19, 20), little attention has been paid to the identification of other possible virulence or fitness factors encoded on the genome of Stx phages. The key feature of Shiga toxin production by E. coli lysogens (STEC) is that Shiga toxin gene expression, and, hence, disease, is a direct consequence of the Stx prophage entering the lytic phase when infectious bacteriophage particles are released along with the toxin itself. The lytic replication cycle of Stx prophages, in common with all known lambdoid bacteriophages, is induced by the autocleavage of the CI repressor, which is driven by the activation of RecA and can be achieved *in vitro* by exposure to UV radiation, mitomycin C, norfloxacin, or other agents that result in DNA damage (21, 22).

There is some evidence that Stx phages have a more profound impact on the E. coli host than merely enabling it to produce Shiga toxin. Preliminary data acquired during signature-tagged mutagenesis experiments identified genes implicated in increased gut adhesion of STEC in calves; of the 59 genes identified, 7 were carried by Stx prophages (23). Microarray analysis of the impact of lysogeny with the Stx2 phage ϕMin27 on the transcriptome of E. coli MG1655 showed upregulation of the expression of 104 genes and downregulation of 62 genes (24). Finally, the lambdoid phage transcriptional regulator CII has been linked to repression of the type III secretion system in EHEC (25), the expression of which is necessary for colonization of the mammalian gut (26).

We previously described the identification of Stx phage genes that are expressed in an E. coli lysogen culture and linked the expression of at least two Stx phage genes of unknown function, *vb_24B_13c* and *res* (15, 27), to the lysogenic state. In any lysogen culture, there is always a background of spontaneous phage induction, which confounds the ability to link the expression of a specific gene with the lysogenic state, and this is further constrained by the sensitivity of the methods used to identify proteins present in low abundance (27). Here we use transcriptome sequencing (RNA-Seq) to produce preliminary gene expression patterns controlled by, or derived from, Stx prophage carriage and determine the influence of one such upregulated operon, glutamic acid decarboxylase (GAD), on the acid resistance phenotype that has been associated with disease-causing Shiga-toxigenic E. coli.

## MATERIALS AND METHODS

### Bacterial strains, growth conditions, and RNA extraction.

E. coli K-12 strain MC1061 was used in all experiments; naive MC1061 refers to cells that have not been infected with bacteriophage ϕ24_B_::Kan (28), and MC1061(ϕ24_B_) refers to MC1061 lysogens of ϕ24_B_::Kan (see Table S1 in the supplemental material). MC1061 and MC1061(ϕ24_B_) were propagated overnight in three biological replicates (16 h), subcultured (1:10), and grown to mid-exponential phase (optical density at 600 nm [OD_600_] of 0.5). Aliquots (1 ml) were harvested, and RNA was extracted by using the RNeasy minikit from Qiagen according to the manufacturer's instructions. These 6 cultures, 3 MC1061 and 3 MC1061(ϕ24_B_) cultures, were then induced with norfloxacin as described previously (27); briefly, cultures were incubated with norfloxacin (1 μg ml^−1^) for 1 h at 37°C with shaking at 200 rpm, followed by dilution of the cultures 1:10 in fresh lysogeny broth, and allowed to recover at 37°C for 40 min with shaking at 200 rpm. Cells were then collected by centrifugation, and RNA was immediately extracted as described above.

### Sequencing of the MC1061(ϕ24_B_) genome.

Prior to the RNA-Seq analysis of the four transcriptomes generated in the study, the genome of MC1061(ϕ24_B_) was sequenced, annotated, and deposited, along with the four transcript libraries, in the European Nucleotide Archive. The genome assembly consists of 5,027,118 bp in 15 scaffolds.

The E. coli MC1061(ϕ24_B_) genome was obtained through whole-genome shotgun pyrosequencing by generating a standard DNA mate-pair library with an 8-kb insert size, using a 454 preparation kit (Roche Applied Sciences, Indianapolis, IN). The genomic DNA (gDNA) sample was sequenced with a GS-FLX system using Titanium chemistry (454 Life Sciences, Roche Applied Sciences). The 454 reads were assembled with Newbler (August 2010 R&D version of GSAssembler; Roche Applied Sciences). The final assembly was annotated on the RAST (Rapid Annotation Using Subsystem Technology) server (29). To improve the quality of the prophage sequence within the lysogen, the 57,677-bp genome of ϕ24_B_ (GenBank accession number HM208303.1) was used to replace the lower-quality prophage sequence residing in the lysogen genome sequence between bp 3129539 and 3186979 (57,440 bp) to form a hybrid genome. All the subsequent analyses and RNA-Seq mapping were done by using the hybrid genome. To compensate for the coordinate numbering following the creation of the hybrid sequence, 236 bp (57,677 bp − 57,440 bp − 1 bp = 236 bp) were added at the starting and ending coordinates of each gene from the original assembly after bp 3186979, while 3,129,538 bp (3,129,539 bp − 1 bp = 3,129,538 bp) were added at the starting and ending coordinates of each gene from the ϕ24_B_ prophage genome.

### Preparation and sequencing of cDNA libraries.

The quantity and quality of harvested RNA were assessed on an Agilent 2100 bioanalyzer using an RNA 6000 Nano Chip kit from Ambion. Equal amounts of RNA were pooled from each biological triplicate to produce four libraries (naive cultures, naive cultures treated with norfloxacin, lysogen cultures, and lysogen cultures treated with norfloxacin). rRNA was depleted from the total RNA samples by using the MICROBExpress bacterial RNA enrichment kit according to the manufacturer's instructions, and the resulting mRNA quantity and quality were evaluated on an Agilent 2100 bioanalyzer using an RNA 6000 Nano Chip kit from Ambion. The RNA was then randomly fragmented by incubation with RNase III (Applied Biosystems) at 37°C for 10 min, recovered by using the RiboMinus concentration module (Invitrogen), and assessed for quantity and size distribution on a bioanalyzer using the RNA 6000 Pico Chip (Agilent). The processed RNA fragments were hybridized and ligated to the adapters from the SOLiD small RNA expression kit (Ambion), and reverse transcription was performed by using ArrayScript reverse transcriptase (Ambion). The resulting cDNA was purified by using the MinElute PCR purification kit (Qiagen). Size selection of cDNA (100 to 200 bp) was performed by using Novex 6% Tris-borate-EDTA (TBE)–urea gels (Invitrogen). The harvested DNA was amplified by using components from the SOLiD small RNA expression kit (Ambion) according to the manufacturer's instructions, and the products were purified by using the PureLink PCR microkit (Invitrogen).

### Data processing and gene expression analysis.

RNA samples as cDNA were sequenced on an ABI SOLiD sequencing platform using v4 chemistry. More than 8 × 10^6^ high-quality, single-end, 50-bp color-space reads were generated per sample. The reads were analyzed by using the ABI BioScope v1.3 whole-transcriptome analysis (WTA) pipeline. The WTA pipeline first aligned the reads onto the E. coli lysogen sequence [MC1061(ϕ24_B_)] and reported the alignments as a BAM file (30). WTA was then used to enumerate reads aligning to each genomic feature in the lysogen genome. Each defined genomic feature was subsequently assigned a tag count, which is the total number of alignments that pass the stringency filters. Finally, the WTA pipeline was used to calculate the normalized level of expression of each genomic feature by using RPKM (reads per kilobase of exon model, per million mapped reads) values (31).

Statistical analysis was performed on normalized read counts for all four samples by using the R platform. The DESeq package was used to analyze differential gene expression across the four libraries (32). The script used in the analysis uses the command cds<-estimateVarianceFunctions(cds, method = “blind”), as recommended in the DESeq manual (32), for processing of samples that are pools and therefore not replicated: table<-read.table(“LN.txt,” header = T), conds<-factor(c[“L,” “N”]), cds<-newCountDataSet(table, conds), cds<-estimateSizeFactors(cds), cds<-estimateVarianceFunctions(cds, method = “blind”), res<-nbinomTest(cds, “L,” “N”), and write.table(res, file = “LNres.txt,” sep = “\t”).

Hierarchical cluster analysis was performed on the base 2 logarithm of gene read counts by using the “qplots” packages in Bioconductor (34).

### Arabinose induction of phage transcriptional regulators.

The ϕ24_B_ genes encoding the regulators CI, CII, and CIII were amplified from MC1061(ϕ24_B_) DNA by using the appropriate PCR primers (see Table S2 in the supplemental material). *cI* was purified and digested with the NcoI and SalI endonucleases. The arabinose-inducible plasmid pBAD/Myc-His (see Table S1 in the supplemental material) was digested with the same enzymes, and both products were cloned to create pϕ24_B_-cI. A construct bearing both *cII* and *cIII* was made by PCR using the primers cII_cIII_phusion and cIII_Sal I (see Table S2 in the supplemental material), digested with NcoI and SalI, and cloned into pBAD/Myc-His to create pϕ24_B_-cIIcIII. MC1061 and MC1061(ϕ24_B_) competent cells were transformed with each of the plasmid constructs, and transformants were selected on LB agar (LBA) containing ampicillin (100 μg ml^−1^).

MC1061 and MC1061(ϕ24_B_) cultures bearing pϕ24_B_-cI or pϕ24_B_-cIIcIII were grown at 37°C to an OD_600_ of ∼0.4, when expression of the cloned phage genes was stimulated by the addition of arabinose (0.01% [wt/vol]), and the cultures were further incubated for 1 h. Production of CI, CII, and CIII was confirmed by SDS-PAGE and Western blot analyses (data not shown).

### Acid resistance assay.

Eight independent cultures of MC1061, MC1061(ϕ24_B_), MC1061/pϕ24_B_-cI, MC1061/pϕ24_B_-cIIcIII, MC1061(ϕ24_B_)/pϕ24_B_-cI, and MC1061(ϕ24_B_)/pϕ24_B_-cIIcIII were grown at 37°C with shaking to an OD_600_ of ∼0.4. The expression of CI, CII, and CIII was induced with arabinose as described above. After 1 h of incubation with arabinose, each culture was diluted 1:10 in fresh LB at pH 2.5 and pH 7.5 and incubated again at 37°C for 2 h. Aliquots of 50 μl from each culture were harvested in triplicate before and after incubation at pH 2.5 and pH 7.5 and serially diluted, and colony counts on LBA were determined. Survival curves were produced for each biological replicate, and statistical analysis was performed by using one-way analysis of variance (ANOVA) followed by a Tukey *post hoc* test; *P* values of <0.05 were considered to be statistically significant.

### Relative RT-qPCR.

RNA samples for reverse transcription-quantitative PCR (RT-qPCR) were prepared, independently, as described above, from three biological replicates of MC1061 and MC1061(ϕ24_B_) cultures. Three biological replicates of MC1061/pϕ24_B_-cI, MC1061/pϕ24_B_-cIIcIII, MC1061(ϕ24_B_)/pϕ24_B_-cI, and MC1061(ϕ24_B_)/pϕ24_B_-cIIcIII cultures were also propagated overnight (16 h), subcultured (1:10), and grown until they reached an OD_600_ of ∼0.4; arabinose (0.01% [wt/vol]) was added to stimulate protein production; and cultures were grown for a further hour. RNA was treated with Turbo DNase (Ambion, TX, USA) according to the manufacturer's instructions. The absence of DNA was verified by qPCR, and the amount of RNA was quantified by using the Nanodrop ND-1000 spectrophotometer (Thermo Fisher Scientific). Each RNA sample (1,000 ng) was reverse transcribed by using random hexamers (Bioline, London, United Kingdom) and the cDNA synthesis kit from Bioline (London, United Kingdom).

RT-qPCR was performed by using a StepOnePlus real-time PCR system (Applied Biosystems); each reaction mixture consisted of 100 ng of cDNA, a 1× SensiFAST SYBR Hi-ROX kit (Bioline, London, United Kingdom), and 200 nM specific primers in a 20-μl reaction mixture. The amplification cycling conditions were as follows: an initial denaturation step at 95°C for 2 min, followed by 39 cycles of denaturation at 95°C for 5 s, annealing at 55°C for 10 s, and extension at 72°C for 5 s. A melting curve analysis was performed for each amplification reaction, with a temperature gradient of 0.1°C from 55°C to 95°C. No-template controls were included in every experiment. The 2^−ΔΔ*CT*^ method (35) was used to calculate relative gene expression levels by using the *pdxA* (4-hydroxythreonine-4-phosphate dehydrogenase) (GenBank accession number NP_414594.1) and *rraB* (RNase E inhibitor) (accession number NP_418676.1) genes as endogenous reference genes. The endogenous reference genes were chosen from among those genes that showed no changes in expression levels across the four samples in the RNA-Seq experiment. All primers used for RT-qPCR are listed in Table S2 in the supplemental material; the efficiency of each primer pair was determined by running calibration curves, in triplicate, against 6 10-fold dilutions (starting with 100 ng) for the PCR amplicons of each target gene that had been cloned into the PCR-Blunt vector (Invitrogen, Paisley, United Kingdom) and linearized with NcoI (NEB, Herts, United Kingdom). Technical replicates were run for every sample, and means were calculated. Statistical analysis was performed on *gadC*, *gadE*, and *gadX* expression data by using one-way ANOVA with a *post hoc* Tukey test. A two-sample *t* test was used to analyze the expression data for the following genes, selected because they have unique sequences amenable to qPCR: *mqo* (GenBank accession number NP_416714.1), *cyoA* (accession number NP_414966), *aceE* (accession number NP_414656.1), *cI* (accession number ADN68413.1), *cro* (accession number ADN68414.1), *vb_24B_19c* (accession number ADN68433.1), *vb_24B_25* (unannotated coding DNA sequence located between nucleotides 33819 and 33905; accession number HM208303.1), *vb_24B_28* (accession number ADN68449.1), and *vb_24B_30* (accession number ADN68451.1). *P* values of <0.05 were considered to be statistically significant.

### Nucleotide sequence accession numbers.

The genome sequences of MC1061(ϕ24_B_) along with the four transcript libraries were deposited in the European Nucleotide Archive, and the accession numbers can be found under BioProject record number PRJEB9491.

## RESULTS

### cDNA sequence output.

The transcriptomes of naive E. coli and isogenic Stx phage lysogen cultures were sequenced. mRNA-derived cDNA libraries were generated from pools comprised of three independent cultures for the following four states: mid-exponential-phase naive and ϕ24_B_ lysogen cultures and norfloxacin-treated naive and ϕ24_B_ lysogen cultures. This enabled sampling of both the stable and induced lysogen transcriptomes for comparison with the naive E. coli host transcriptome with and without norfloxacin treatment. Reads (82.8 million to 87.3 million; 50 bp in length) were obtained from each of the four pooled samples, with 57 to 65% of reads mapping to the E. coli MC1061 genome with or without the ϕ24_B_ prophage incorporated at the primary insertion site (36). A summary analysis of the cDNA sequence output is presented in Table 1.

**TABLE 1:** Summary of cDNA sequencing output

Sample	Reads generated (bp)	% mapped reads	% uniquely mapped reads^*a*^	Coverage (Mb)
MC1061(ϕ24_B_)	82,804,709	58.29	37.51	2,228.74
MC1061	86,132,244	64.7	45.83	2,632.59
MC1061(ϕ24_B_), induced	82,469,423	58.11	40.41	2,247.81
MC1061, induced	87,298,027	57.15	34.9	2,293.59

a Zero mismatches.

### Gene expression in response to norfloxacin treatment and expression of ϕ24_B_ bacteriophage genes.

Prophage gene expression analysis must take account of the effect of ongoing spontaneous induction of the prophage in the bacterial lysogen culture, which may occur at rates as high as 1:1,000 cells. We have demonstrated that this can be achieved by using comparative data sets generated by exogenous prophage induction, as previously demonstrated with RT-qPCR gene expression profiling (27). MC1061 and MC1061(ϕ24_B_) samples treated with norfloxacin, to activate the lytic cycle via the SOS response, were included here to enable us to properly identify gene expression changes that can be ascribed to prophage carriage on the E. coli chromosome. Additionally, and of particular interest to this study, by using these controls, we were able to identify phage-carried genes that are potentially expressed during the lysogenic cycle.

The overall pattern of gene expression in response to norfloxacin treatment is depicted in Fig. S1 in the supplemental material and can be summarized as a significant upregulation of genes for DNA repair and iron and phosphate acquisition concomitant with a downregulation of genes and operons involved in carbon and nitrogen metabolism, energy generation, and motility (see Table S3 in the supplemental material). Upregulation of the iron and phosphate acquisition operons/genes has been linked to prophage carriage by a previous microarray-based study of Stx phage ϕMin27 lysogens (24). In that microarray study, controls for the effect of spontaneous prophage induction were not included, and our data indicate that comparative upregulation in a lysogen culture is actually due to large gene expression changes in the small subset of the population undergoing spontaneous induction (see Table S3 in the supplemental material). The impact of significant gene expression profile changes on the small subset of cells in a lysogen culture undergoing spontaneous induction was described previously for E. coli populations harboring the ϕ24_B_ prophage (27).

There is always a basal level of expression of bacteriophage genes associated with the lytic phage replication cycle in any lysogen culture due to some proportion of that culture undergoing spontaneous induction (27). In order to establish whether a phage gene is truly expressed during lysogeny or as part of the lytic cycle, it is necessary to examine the expression of the gene upon induction. Here the expression levels of 68 of the 93 ϕ24_B_ genes increased >20-fold upon prophage induction into the lytic cycle, although for 17 of these genes, DESeq analysis did not classify the upregulation as significant (see Table S4 in the supplemental material). The expression levels of 14 genes were unaltered, and these genes include *cI*, responsible for the maintenance of the lysogenic state; *int*, the phage integrase which has been shown to be uncoupled from the phage regulatory network in ϕ24_B_ (37); *bor*, characterized in bacteriophage lambda (38); *stk*, a serine-threonine kinase; and 10 genes of unknown function (see Table S4 in the supplemental material), 2 of which, *vb_24B_13c* and *res*, were previously been shown to be expressed in E. coli ϕ24_B_ lysogens (27).

**FIG 1:**
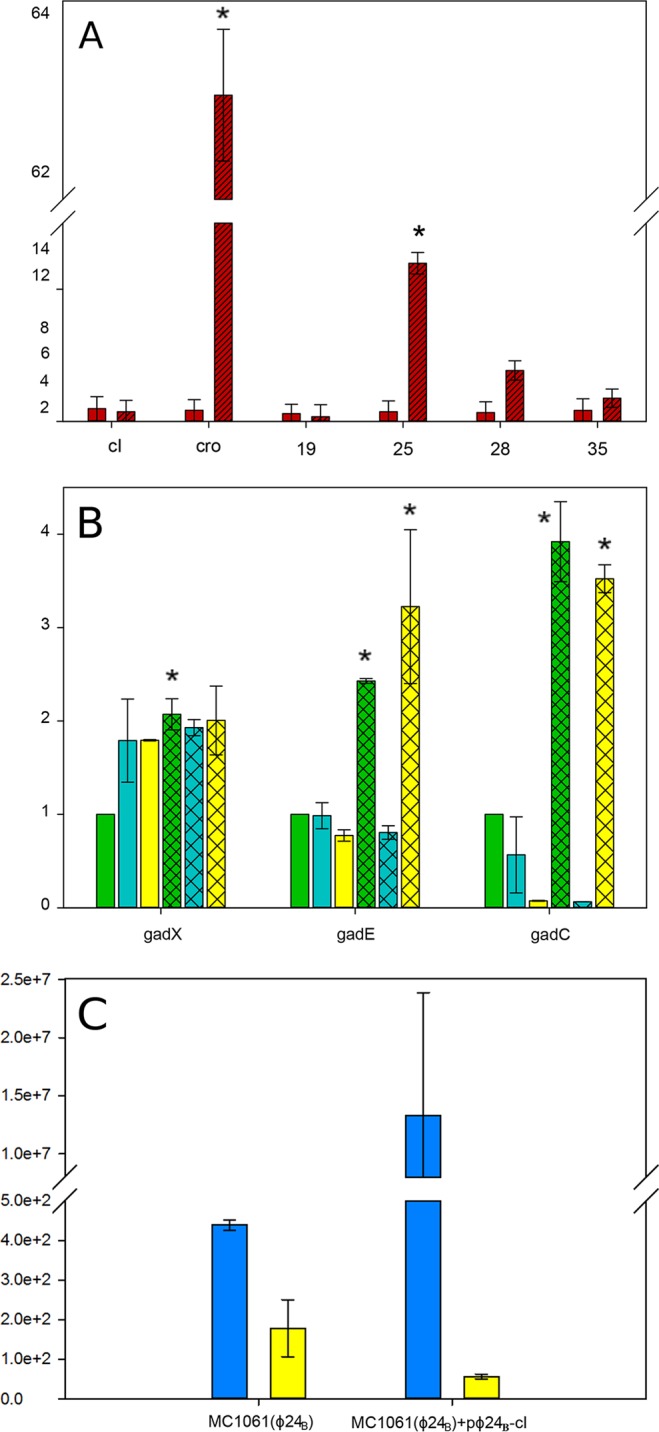
(A) Fold changes in expression of the ϕ24_B_ genes *cI*, *cro*, *vb_24B_19c*, *vb_24B_25*, *vb_24B_28*, and *vb_24B_30* determined by RT-qPCR. Solid red bars, MC1061(ϕ24_B_); hatched red bars, induced MC1061(ϕ24_B_). Error bars represent standard errors of the means (*n* = 3). * represents statistically significant values in a two-sample *t* test (*P* value of <0.05). (B) *gadX*, *gadE*, and *gadC*. Expression levels were normalized against the values for the endogenous reference genes *pdxA* and *rraB*. Solid green bars, MC1061; hatched green bars, MC1061(ϕ24_B_); solid blue bars, MC1061/pϕ24_B_-cI; hatched blue bars, MC1061(ϕ24_B_)/pϕ24_B_-cI; solid yellow bars, MC1061/pϕ24_B_-cIIcIII; hatched yellow bars, MC1061(ϕ24_B_)/pϕ24_B_-cIIcIII. Error bars represent standard errors of the means (*n* = 3). * represents statistically significant values compared to MC1061, as determined by one-way ANOVA with a *post hoc* Tukey test (*P* value of <0.05). (C) Expression levels of the ϕ24_B_ genes *cI* (blue) and *cII* (yellow) in MC1061(ϕ24_B_) and MC1061(ϕ24_B_)/pϕ24_B_-cI after incubation with 0.01% arabinose. Error bars represent standard errors of the means (*n* = 3).

Four phage genes (*vb_24B_19c*, *vb_24B_25*, *vb_24B_28*, and *vb_24B_30*) were selected to validate the RNA-Seq data. The regulatory genes *cI* and *cro* were included as controls, and relative RT-qPCR was performed as described in Materials and Methods. These data are presented in Fig. 1A. In accordance with data from the transcriptomic analysis, expression levels of *cI*, *vb_24B_19c*, *vb_24B_28*, and *vb_24B_30* did not change significantly, while the expression level of *vb_24B_25* showed a significant 10-fold increase upon induction (*P* value of <0.05). The latter expression level is far short of the increase of >60-fold (*P* value of <0.005) for *cro*, the control for gene expression linked to bacteriophage induction. This qPCR-derived value for *cro* is in agreement with the 45-fold increase observed in the RNA-Seq data (see Table S4 in the supplemental material).

### Gene expression changes associated with Stx prophage carriage.

Comparison of the MC1061 and MC1061(ϕ24_B_) transcriptomes revealed that the majority of bacterial expression changes detected are likely to be simply a consequence of the SOS response occurring in the MC1061(ϕ24_B_) sample (see Table S3 in the supplemental material). In most cases, the decreased gene expression in the lysogen can be ascribed to spontaneous bacteriophage induction. However, there were several genes that appear to be truly downregulated in the lysogen, i.e., due to phage carriage. *mqo* encodes a malate:quinone oxidoreductase that was downregulated 5-fold in the lysogen sample but showed slightly increased expression levels in both lysogen and naive samples treated with norfloxacin (Table 2). Similarly, the expression levels of *aceEF* were significantly decreased (Table 2); *aceEF* encode two of the subunits that form pyruvate dehydrogenase, the enzyme that provides acetyl coenzyme A (acetyl-CoA) for the tricarboxylic acid cycle (39, 40). The expression level of the gene encoding the third subunit of pyruvate dehydrogenase, *aceK*, was reduced 1.7-fold. The expression level of the *cyoABCDE* operon was also found to be reduced in the lysogen; these genes encode the subunits of the cytochrome *b* oxidase, one of the three major terminal oxidases in the aerobic respiratory chain of E. coli. These observations based on RNA-Seq analysis of pooled samples require independent verification, and this is supplied by the RT-qPCR data for *aceK*, *mqo*, and *cyoA* (see Fig. S2 in the supplemental material), which support the RNA-Seq observations.

**TABLE 2:** Bacterial genes differentially expressed between MC1061 and MC1061(ϕ24_B_) before norfloxacin treatment^*a*^

Gene	Function^*b*^	Fold change	*P* value
Genes downregulated in MC1061(ϕ24_B_)			
*cyoB*	Cytochrome O ubiquinol oxidase	3.41	0.001
*cyoA*	Cytochrome O ubiquinol oxidase	3.78	0.000
*cyoC*	Cytochrome O ubiquinol oxidase	3.12	0.001
*cyoD*	Cytochrome O ubiquinol oxidase	3.12	0.002
*cyoE*	Heme O synthase, protoheme IX farnesyltransferase	2.1	0.022
*aceE*	Pyruvate dehydrogenase E1 component	3.24	0.001
*aceF*	Pyruvate dehydrogenase E2 component	2.40	0.011
Genes upregulated in MC1061(ϕ24_B_)			
*hdeA*	Chaperone HdeA	3.004	0.038
*hdeD*	Membrane transporter, H-NS repressed	3.127	0.044
*rpoS*	Sigma transcription factor controlling a regulon of genes required for protection against external stresses	3.427	0.021
*gadA*	Glutamate decarboxylase	3.558	0.043
*gadB*	Glutamate decarboxylase	3.133	0.030
*gadC*	Probable glutamate/gamma-aminobutyrate antiporter	3.414	0.021
*gadE*	Transcriptional activator	5.989	0.032
*gadW*	HTH-type transcriptional regulator	5.196	0.000
*gadX*	HTH-type transcriptional regulator	3.115	0.004
*fimF*	Minor component of type 1 fimbriae	6.543	0.028
*fimA*	Major subunit of type 1 subunit fimbriae	19.451	0.000
*fimC*	Required for biogenesis of type 1 fimbriae	17.396	0.000
*fimD*	Type 1 fimbria anchoring protein involved in export and assembly of *fimA* fimbrial subunits across the outer membrane	11.850	0.000
*fimE*	Type 1 fimbria regulatory protein	21.938	0.022
*fimG*	Type 1 fimbria adapter subunit	9.526	0.014
*fimI*	Type 1 fimbria protein	20.345	0.000

a Excludes ϕ24_B_ genes that are presented in Table S4 in the supplemental material.

b HTH, helix-turn-helix.

Among the lysogen-upregulated genes were operons encoding type I fimbriae and the GAD acid stress island (Table 2). The *fim* operon comprises 9 genes, 7 of which (*fimACDEFGI*) showed significantly increased expression in the lysogen. The genes comprising the GAD acid stress island control the glutamate-dependent acid resistance mechanism in E. coli (41–43). The *gadAB* genes encode two glutamate decarboxylases that, together with *gadC*, comprise the structural genes of the GAD operon. Expression of the *gadABC* genes is under the control of the global regulator *gadE* and two additional regulators, *gadX* and *gadW*. All of these genes were significantly upregulated in the lysogen (Table 2).

### Increased acid resistance of MC1061(ϕ24_B_).

**FIG 2:**
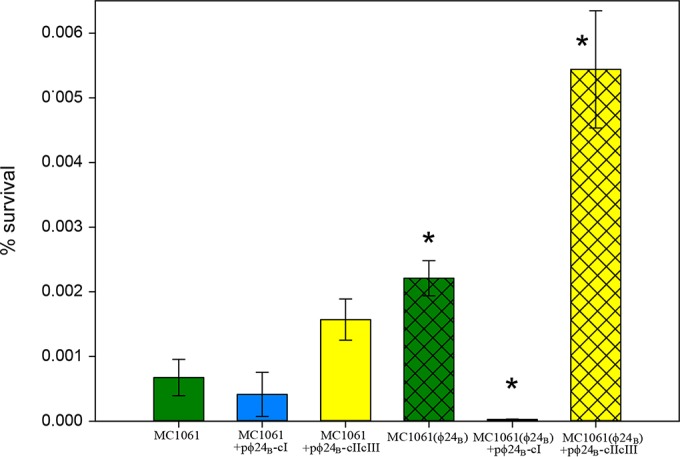
Survival of MC1061, MC1061/pϕ24_B_-cI, MC1061/pϕ24_B_-cIIcIII, MC1061(ϕ24_B_), MC1061(ϕ24_B_)/pϕ24_B_-cI, and MC1061(ϕ24_B_)/pϕ24_B_-cIIcIII after incubation in LB at pH 2.5. Error bars represent standard errors of the means (*n* = 8). * represents statistically significant values compared to MC1061, as determined by one-way ANOVA with a *post hoc* Tukey test (*P* value of <0.05).

Among the data in Table 2, the 3- to 5-fold-increased expression levels of components of the GAD system, which controls acid resistance in E. coli, are particularly striking. Acid resistance is a well-studied feature of the biology of E. coli O157 (43), and the low infective dose characteristic of this pathogenic serotype is thought to be related to its ability to survive passage through the stomach. To assess whether ϕ24_B_ imparts increased acid resistance to the lysogen via upregulation of the GAD system, both MC1061 and MC1061(ϕ24_B_) were incubated at pH 2.5 for a period of 2 h at 37°C, and survival was determined (Fig. 2). The rate of survival of the lysogen MC1061(ϕ24_B_) under acidic conditions was reproducibly ∼3-fold higher than that of the naive strain, MC1061 (*P* value of <0.05). In order to determine if phage regulator proteins that control phage gene transcription (CI, CII, and CIII) were involved in activating the GAD system, the genes for these proteins were cloned into MC1061, which was then subjected to a 2-h pH 2.5 challenge after induction of gene expression. The cloning vector was an arabinose-inducible expression plasmid modified to harbor either the *cI* gene or both the *cII* and *cIII* genes, pϕ24_B_-cI or pϕ24_B_-cIIcIII, respectively. CI is the transcriptional repressor that maintains lysogeny (44). CII is a transcriptional regulator crucial to the lysis-lysogeny decision during the early stage of the phage life cycle, and it is rapidly degraded by the bacterial FtsH protease (44–46). The CIII protein acts by inhibiting FtsH, indirectly protecting CII from degradation (45).

MC1061 harboring pϕ24_B_-cI possessed the same acid survival phenotype as that of naive cells (Fig. 2). In contrast, the MC1061 clone expressing both CII and CIII was more acid resistant than the lysogen (Fig. 2). In order to determine the impact of CII-CIII expression on *gadE*, *gadX*, and *gadC*, the regulators of the acid resistance GAD system in E. coli, RT-qPCR data were generated by using MC1061 and MC1061(ϕ24_B_) mid-exponential-phase cultures. The relative expression levels of *gadE*, *gadX*, and *gadC* were calculated by using the naive MC1061 sample as the calibrator (Fig. 1B). The *gadX* expression level was significantly higher in the MC1061(ϕ24_B_) samples than in the MC1061 samples (*P* value of <0.05), but this gene exhibited a very similar relative expression profile in all other constructs and strains (Fig. 1B). The expression of *gadE* was also increased in MC1061(ϕ24_B_) samples with respect to MC1061; however, there was no significant effect on *gadE* expression due to the presence of CI or CII-CIII in MC1061. There was a reduction in *gadE* expression in MC1061(ϕ24_B_)/pϕ24_B_-cI and an increase in expression in MC1061(ϕ24_B_)/pϕ24_B_-cIIcIII compared to MC1061(ϕ24_B_) (Fig. 1B). Interestingly, the most pronounced effect was observed for *gadC* expression. There was a very significant difference between the MC1061(ϕ24_B_) and MC1061 samples (*P* value of <0.01), but expression was significantly reduced by the production of CI in MC1061(ϕ24_B_), while the production of CII-CIII maintained high levels of *gadC* expression (Fig. 1B). GadC is one of the proteins directly involved in acid resistance (membrane transporter of glutamate) rather than functioning as a regulator.

In bacteriophage lambda, CII binds to a well-defined sequence, a tetranucleotide repeat (TTGCN_6_TTGC) flanking the −35 region of the promoters for P_RE_, P_I_, and P_aQ_, controlling the ability of lambda to establish CI production and to express integrase and the anti-Q transcript, respectively (47–49). In ϕ24_B_, there is a single nucleotide change in this repeat in the same locations, but the GAD operon had no matches to either of the CII binding sequences, suggesting that the substantial effect of CII_ϕ24B_ on acid resistance is pleiotropic.

The confounding issue with CII increasing acid resistance is that CI (repressor) should turn off all CII expression in the lysogen. We determined the levels of the *cII* transcript in MC1061(ϕ24_B_) cultures by RT-qPCR and demonstrated that measurable numbers of transcripts were present, corroborating our RNA-Seq data (Fig. 1C). We then measured the levels of the *cII* transcript in MC1061(ϕ24_B_)/pϕ24_B_-cI cultures grown in the presence of 0.01% arabinose by RT-qPCR and demonstrated a 3-fold decrease in *cII* transcript levels (Fig. 1C). These data, combined with the observations that the CI expression level is very low (Fig. 1A) in the lysogen and that it is known that the operator binding sites in ϕ24_B_ and related phages like 933W are missing or reside in the cI open reading frame (50, 51), support our contention that CII is present and active in the lysogen. Furthermore, we were able to reestablish acid sensitivity in the ϕ24_B_ lysogen by overexpressing *cI* (Fig. 2); overexpression of *cI* has also been shown to abolish CII downregulation of a type III secretion system in a Shiga-toxigenic E. coli lysogen (EDL933W) (25). We made a ϕ24_B_ lysogen of strain TUV93-0 (EDL933 with both Shiga-toxigenic phages removed) and compared the acid resistances of the naive and lysogen strains. We were able to replicate our observations with MC1061; i.e., the levels of acid resistance were 4-fold higher in the lysogen and >4-fold higher in a TUV93 lysogen (data not shown).

## DISCUSSION

Sequencing of the transcriptomes of E. coli MC1061 and MC1061(ϕ24_B_) is an invaluable general tool to discern the impact that a bacteriophage can have on its host transcriptome as well as to determine which bacteriophage genes are expressed during the lysogenic state.

Shiga toxin-encoding lambdoid phages were discovered in 1983 (52), and while their contribution to the pathogenic profile of Shiga-toxigenic E. coli has been well documented, their impact on the biology of the E. coli host has not been fully characterized. A microarray-based comparison of gene expression levels in a naive strain and a strain harboring a Shiga-toxigenic phage (ϕMin27) identified groups of genes that were up- or downregulated (24), among them the GAD operon, also identified here as being particularly responsive to lysogeny. We describe the application of RNA-Seq technology to the relative quantification of gene expression in naive and lysogenic E. coli strains, addressing genes carried by both the ϕ24_B_ phage and the host and crucially correcting for transcriptional changes that are due to phage induction via the SOS response. It is important to differentiate gene expression patterns that are due to spontaneous induction in a lysogen culture from the carriage and expression of the prophage genes *per se*. Hence, here we can identify genes involved in iron acquisition, phosphate metabolism, carbohydrate metabolism, and anaerobic respiration (see Table S3 in the supplemental material) that are in this category, although further analysis is required to confirm their role in the SOS response.

Bacteriophage lambda carries *lom* and *bor*, two genes that are expressed in E. coli and enhance the lysogen's adhesion to epithelial cells and resistance to immune cell attack (38, 53). These genes are also carried by ϕ24_B_, and expression by the lysogen was previously described as being constitutive and uncoupled from the phage regulatory network (27). The RNA-Seq data here fully support that conclusion. The phenomenon represented by prophage-controlled acid resistance, i.e., the control of host cell genes by phage regulators resulting in the alteration of a fitness/virulence trait, was described previously (24), but the mechanism by which the prophage effects this change is addressed here. Our data (from RNA-Seq, RT-qPCR, and acid resistance assays) have demonstrated that the phage-encoded CII transcriptional activator controls the acid response in the lysogen. There are four distinct acid resistance mechanisms described for E. coli (54, 55), and the glutamate-dependent acid resistance system is the most efficient of the four. GAD has long been recognized as a virulence factor in EHEC that contributes to its low infective dose by helping it survive exposure to pHs as low as 1.5 (56, 57).

**FIG 3:**
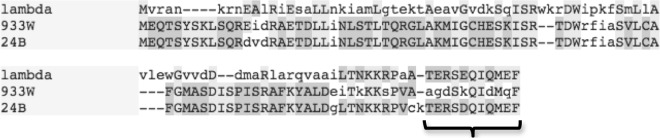
Shared homology across CII proteins from three lambdoid phages. The T-Coffee multialignment program hosted by the EBI (http://www.ebi.ac.uk/Tools/msa/tcoffee/) was used to analyze homology shared between CII proteins from phages lambda, 933W, and ϕ24_B_. The CII proteins encoded by the Stx phages 933W and ϕ24_B_ share significant levels of homology, except for their C termini. The C-terminal residues shared between proteins encoded by lambda phage and ϕ24_B_ (highlighted by a bracket) serve as the FtsH recognition site and therefore control the half-life of CII.

CII from 933W has also been assigned a role in controlling, specifically decreasing, the expression of a type III secretion system, another virulence factor of EHEC (25), while prophages that encode regulators that upregulate type III secretion have also been found (58). The CII protein of ϕ24_B_ shares significant homology with the CII protein of 933W. However, these two proteins differ at their carboxyl termini (Fig. 3). In fact, the carboxyl terminus of ϕ24_B_ shares considerable homology with CII encoded by bacteriophage lambda; this shared sequence is in fact the domain that targets CII for destruction by the cellular protease FtsH. Thus, the CII proteins of ϕ24_B_ and 933W would be expected to share similar transcriptional regulatory functions without sharing susceptibility to FtsH destruction, while the CII protein encoded by lambda should not have the same phenotypic effects.

The impact of CII expression and its control of the survival of Stx phage lysogens in transit through the gastric system still need to be addressed through animal experimentation, but our data suggest that CII will directly improve the survival of E. coli in the acidic environment of the stomach. Significantly, we were able to introduce ϕ24_B_ into an E. coli O157-derived strain and demonstrate increased acid resistance in the lysogen. Our data point to the fact that the Stx phages themselves directly contribute to the fitness of their lysogens and that it is not simply the carriage of *stx* genes that provides a selective advantage for the dissemination and host range expansion that has been seen since the first E. coli O157:H7 outbreak in 1982.

## Supplementary Material

Supplemental material

## References

[1] Canchaya C, Fournous G, Brüssow H 2004 The impact of prophages on bacterial chromosomes. Mol Microbiol 53:9–18. doi:10.1111/j.1365-2958.2004.04113.x. 15225299

[2] Fortier L-C, Sekulovic O 2013 Importance of prophages to evolution and virulence of bacterial pathogens. Virulence 4:354–365. doi:10.4161/viru.24498. 23611873 PMC3714127

[3] Zhang Y, Laing C, Steele M, Ziebell K, Johnson R, Benson AK, Taboada E, Gannon VPJ 2007 Genome evolution in major Escherichia coli O157:H7 lineages. BMC Genomics 8:121. doi:10.1186/1471-2164-8-121. 17506902 PMC1890555

[4] Ohnishi M, Kurokawa K, Hayashi T 2001 Diversification of Escherichia coli genomes: are bacteriophages the major contributors? Trends Microbiol 9:481–485. doi:10.1016/S0966-842X(01)02173-4. 11597449

[5] Brüssow H, Canchaya C, Hardt W-D 2004 Phages and the evolution of bacterial pathogens: from genomic rearrangements to lysogenic conversion. Microbiol Mol Biol Rev 68:560–602. doi:10.1128/MMBR.68.3.560-602.2004. 15353570 PMC515249

[6] Boyd EF, Brüssow H 2002 Common themes among bacteriophage-encoded virulence factors and diversity among the bacteriophages involved. Trends Microbiol 10:521–529. doi:10.1016/S0966-842X(02)02459-9. 12419617

[7] Allison HE 2007 Stx-phages: drivers and mediators of the evolution of STEC and STEC-like pathogens. Future Microbiol 2:165–174. doi:10.2217/17460913.2.2.165. 17661653

[8] Figueroa-Bossi N, Uzzau S, Maloriol D, Bossi L 2001 Variable assortment of prophages provides a transferable repertoire of pathogenic determinants in Salmonella. Mol Microbiol 39:260–271. doi:10.1046/j.1365-2958.2001.02234.x. 11136448

[9] Bae T, Baba T, Hiramatsu K, Schneewind O 2006 Prophages of Staphylococcus aureus Newman and their contribution to virulence. Mol Microbiol 62:1035–1047. doi:10.1111/j.1365-2958.2006.05441.x. 17078814

[10] Feng P, Lampel KA, Karch H, Whittam TS 1998 Genotypic and phenotypic changes in the emergence of Escherichia coli O157:H7. J Infect Dis 177:1750–1753. doi:10.1086/517438. 9607864

[11] Wick LM, Qi W, Lacher DW, Whittam TS 2005 Evolution of genomic content in the stepwise emergence of Escherichia coli O157:H7. J Bacteriol 187:1783–1791. doi:10.1128/JB.187.5.1783-1791.2005. 15716450 PMC1064018

[12] Riley LW, Remis RS, Helgerson SD, McGee HB, Wells JG, Davis BR, Hebert RJ, Olcott ES, Johnson LM, Hargrett NT, Blake PA, Cohen ML 1983 Hemorrhagic colitis associated with a rare Escherichia coli serotype. N Engl J Med 308:681–685. doi:10.1056/NEJM198303243081203. 6338386

[13] Griffin PM, Ostroff SM, Tauxe RV, Greene KD, Wells JG, Lewis JH, Blake PA 1988 Illnesses associated with Escherichia coli O157:H7 infections. A broad clinical spectrum. Ann Intern Med 109:705–712. 3056169 10.7326/0003-4819-109-9-705

[14] Karmali MA, Petric M, Lim C, Fleming PC, Steele BT 1983 Escherichia coli cytotoxin, haemolytic-uraemic syndrome, and haemorrhagic colitis. Lancet ii:1299–1300. 6139632 10.1016/s0140-6736(83)91167-4

[15] Smith DL, Rooks DJ, Fogg PCM, Darby AC, Thomson NR, McCarthy AJ, Allison HE 2012 Comparative genomics of Shiga toxin encoding bacteriophages. BMC Genomics 13:311. doi:10.1186/1471-2164-13-311. 22799768 PMC3430580

[16] Bergan J, Dyve Lingelem AB, Simm R, Skotland T, Sandvig K 2012 Shiga toxins. Toxicon 60:1085–1107. doi:10.1016/j.toxicon.2012.07.016. 22960449

[17] Tesh VL 2010 Induction of apoptosis by Shiga toxins. Future Microbiol 5:431–453. doi:10.2217/fmb.10.4. 20210553 PMC2855686

[18] Mauro SA, Koudelka GB 2011 Shiga toxin: expression, distribution, and its role in the environment. Toxins 3:608–625. doi:10.3390/toxins3060608. 22069728 PMC3202840

[19] Lainhart W, Stolfa G, Koudelka GB 2009 Shiga toxin as a bacterial defense against a eukaryotic predator, Tetrahymena thermophila. J Bacteriol 191:5116–5122. doi:10.1128/JB.00508-09. 19502393 PMC2725575

[20] Steinberg KM, Levin BR 2007 Grazing protozoa and the evolution of the Escherichia coli O157:H7 Shiga toxin-encoding prophage. Proc Biol Sci 274:1921–1929. doi:10.1098/rspb.2007.0245. 17535798 PMC2211389

[21] Loś JM, Loś M, Węgrzyn G, Wegrzyn A 2009 Differential efficiency of induction of various lambdoid prophages responsible for production of Shiga toxins in response to different induction agents. Microb Pathog 47:289–298. doi:10.1016/j.micpath.2009.09.006. 19761828

[22] Little JW 1984 Autodigestion of LexA and phage lambda repressors. Proc Natl Acad Sci U S A 81:1375–1379. doi:10.1073/pnas.81.5.1375. 6231641 PMC344836

[23] Dziva F, van Diemen PM, Stevens MP, Smith AJ, Wallis TS 2004 Identification of Escherichia coli O157:H7 genes influencing colonization of the bovine gastrointestinal tract using signature-tagged mutagenesis. Microbiology 150:3631–3645. doi:10.1099/mic.0.27448-0. 15528651

[24] Su LK, Lu CP, Wang Y, Cao DM, Sun JH, Yan YX 2010 Lysogenic infection of a Shiga toxin 2-converting bacteriophage changes host gene expression, enhances host acid resistance and motility. Mol Biol 44:54–66. doi:10.1134/S0026893310010085. 20198860

[25] Xu X, McAteer SP, Tree JJ, Shaw DJ, Wolfson EBK, Beatson SA, Roe AJ, Allison LJ, Chase-Topping ME, Mahajan A, Tozzoli R, Woolhouse MEJ, Morabito S, Gally DL 2012 Lysogeny with Shiga toxin 2-encoding bacteriophages represses type III secretion in enterohemorrhagic Escherichia coli. PLoS Pathog 8:e1002672. doi:10.1371/journal.ppat.1002672. 22615557 PMC3355084

[26] Naylor SW, Roe AJ, Nart P, Spears K, Smith DGE, Low JC, Gally DL 2005 Escherichia coli O157:H7 forms attaching and effacing lesions at the terminal rectum of cattle and colonization requires the LEE4 operon. Microbiology 151:2773–2781. doi:10.1099/mic.0.28060-0. 16079353

[27] Riley LM, Veses-Garcia M, Hillman JD, Handfield M, McCarthy AJ, Allison HE 2012 Identification of genes expressed in cultures of E. coli lysogens carrying the Shiga toxin-encoding prophage Phi24B. BMC Microbiol 12:42. doi:10.1186/1471-2180-12-42. 22439817 PMC3342100

[28] James CE, Stanley KN, Allison HE, Flint HJ, Stewart CS, Sharp RJ, Saunders JR, McCarthy AJ 2001 Lytic and lysogenic infection of diverse Escherichia coli and Shigella strains with a verocytotoxigenic bacteriophage. Appl Environ Microbiol 67:4335–4337. doi:10.1128/AEM.67.9.4335-4337.2001. 11526041 PMC93165

[29] Aziz RK, Bartels D, Best AA, DeJongh M, Disz T, Edwards RA, Formsma K, Gerdes S, Glass EM, Kubal M, Meyer F, Olsen GJ, Olson R, Osterman AL, Overbeek RA, McNeil LK, Paarmann D, Paczian T, Parrello B, Pusch GD, Reich C, Stevens R, Vassieva O, Vonstein V, Wilke A, Zagnitko O 2008 The RAST server: rapid annotations using subsystems technology. BMC Genomics 9:75. doi:10.1186/1471-2164-9-75. 18261238 PMC2265698

[30] Li H, Handsaker B, Wysoker A, Fennell T, Ruan J, Homer N, Marth G, Abecasis G, Durbin R, 1000 Genome Project Data Processing Subgroup. 2009 The Sequence Alignment/Map format and SAMtools. Bioinformatics 25:2078–2079. doi:10.1093/bioinformatics/btp352. 19505943 PMC2723002

[31] Mortazavi A, Williams BA, McCue K, Schaeffer L, Wold B 2008 Mapping and quantifying mammalian transcriptomes by RNA-Seq. Nat Methods 5:621–628. doi:10.1038/nmeth.1226. 18516045 PMC13303166

[32] Anders S, Huber W 2010 Differential expression analysis for sequence count data. Genome Biol 11:R106. doi:10.1186/gb-2010-11-10-r106. 20979621 PMC3218662

[33] Reference deleted.

[34] Gentleman RC, Carey VJ, Bates DM, Bolstad B, Dettling M, Dudoit S, Ellis B, Gautier L, Ge Y, Gentry J, Hornik K, Hothorn T, Huber W, Iacus S, Irizarry R, Leisch F, Li C, Maechler M, Rossini AJ, Sawitzki G, Smith C, Smyth G, Tierney L, Yang JY, Zhang J 2004 Bioconductor: open software development for computational biology and bioinformatics. Genome Biol 5:R80. doi:10.1186/gb-2004-5-10-r80. 15461798 PMC545600

[35] Arocho A, Chen B, Ladanyi M, Pan Q 2006 Validation of the 2-DeltaDeltaCt calculation as an alternate method of data analysis for quantitative PCR of BCR-ABL P210 transcripts. Diagn Mol Pathol 15:56–61. doi:10.1097/00019606-200603000-00009. 16531770

[36] Fogg PCM, Gossage SM, Smith DL, Saunders JR, McCarthy AJ, Allison HE 2007 Identification of multiple integration sites for Stx-phage Phi24B in the Escherichia coli genome, description of a novel integrase and evidence for a functional anti-repressor. Microbiology 153:4098–4110. doi:10.1099/mic.0.2007/011205-0. 18048923

[37] Fogg PCM, Rigden DJ, Saunders JR, McCarthy AJ, Allison HE 2011 Characterization of the relationship between integrase, excisionase and antirepressor activities associated with a superinfecting Shiga toxin encoding bacteriophage. Nucleic Acids Res 39:2116–2129. doi:10.1093/nar/gkq923. 21062824 PMC3064807

[38] Barondess JJ, Beckwith J 1990 A bacterial virulence determinant encoded by lysogenic coliphage lambda. Nature 346:871–874. doi:10.1038/346871a0. 2144037

[39] Schwartz ER, Old LO, Reed LJ 1968 Regulatory properties of pyruvate dehydrogenase from Escherichia coli. Biochem Biophys Res Commun 31:495–500. doi:10.1016/0006-291X(68)90504-4. 4871329

[40] Spencer ME, Guest JR 1985 Transcription analysis of the *sucAB*, *aceEF* and *lpd* genes of Escherichia coli. Mol Gen Genet 200:145–154. doi:10.1007/BF00383328. 3897791

[41] Ma Z, Gong S, Richard H, Tucker DL, Conway T, Foster JW 2003 GadE (YhiE) activates glutamate decarboxylase-dependent acid resistance in Escherichia coli K-12. Mol Microbiol 49:1309–1320. doi:10.1046/j.1365-2958.2003.03633.x. 12940989

[42] Hommais F 2004 GadE (YhiE): a novel activator involved in the response to acid environment in Escherichia coli. Microbiology 150:61–72. doi:10.1099/mic.0.26659-0. 14702398

[43] Kailasan Vanaja S, Bergholz TM, Whittam TS 2009 Characterization of the Escherichia coli O157:H7 Sakai GadE regulon. J Bacteriol 191:1868–1877. doi:10.1128/JB.01481-08. 19114477 PMC2648353

[44] Ptashne M, Jeffrey A, Johnson AD, Maurer R, Meyer BJ, Pabo CO, Roberts TM, Sauer RT 1980 How the λ repressor and cro work. Cell 19:1–11. doi:10.1016/0092-8674(80)90383-9. 6444544

[45] Kobiler O, Rokney A, Oppenheim AB 2007 Phage lambda CIII: a protease inhibitor regulating the lysis-lysogeny decision. PLoS One 2:e363. doi:10.1371/journal.pone.0000363. 17426811 PMC1838920

[46] Oppenheim AB, Kobiler O, Stavans J, Court DL, Adhya S 2005 Switches in bacteriophage lambda development. Genetics 39:409–429. doi:10.1146/annurev.genet.39.073003.113656. 16285866

[47] Shih M-C, Gussin GN 1983 Differential effects of mutations on discrete steps in transcription initiation at the λ P_RE_ promoter. Cell 34:941–949. doi:10.1016/0092-8674(83)90551-2. 6226364

[48] Place N, Fien K, Mahoney ME, Wulff DL, Ho YS, Debouck C, Rosenberg M, Shih MC, Gussin GN 1984 Mutations that alter the DNA binding site for the bacteriophage lambda cII protein and affect the translation efficiency of the *cII* gene. J Mol Biol 180:865–880. doi:10.1016/0022-2836(84)90261-4. 6241264

[49] Simatake H, Rosenberg M 1981 Purified lambda regulatory protein cII positively activates promoters for lysogenic development. Nature 292:128–132. doi:10.1038/292128a0. 6264321

[50] Waldor MK, Friedman DI 2005 Phage regulatory circuits and virulence gene expression. Curr Opin Microbiol 8:459–465. doi:10.1016/j.mib.2005.06.001. 15979389

[51] Bullwinkle TJ, Koudelka GB 2011 The lysis-lysogeny decision of bacteriophage 933W: a 933W repressor-mediated long-distance loop has no role in regulating 933W P_RM_ activity. J Bacteriol 193:3313–3323. doi:10.1128/JB.00119-11. 21551291 PMC3133280

[52] O'Brien AD, Newland JW, Miller SF, Holmes RK, Smith HW, Formal SB 1984 Shiga-like toxin-converting phages from Escherichia coli strains that cause hemorrhagic colitis or infantile diarrhea. Science 226:694–696. doi:10.1126/science.6387911. 6387911

[53] Vica Pacheco S, González OG, Contreras GLP 1997 The *lom* gene of bacteriophage λ is involved in Escherichia coli K12 adhesion to human buccal epithelial cells. FEMS Microbiol Lett 156:129–132. doi:10.1016/S0378-1097(97)00415-1. 9368371

[54] Castanie-Cornet MP, Cam K, Bastiat B, Cros A, Bordes P, Gutierrez C 2010 Acid stress response in Escherichia coli: mechanism of regulation of *gadA* transcription by RcsB and GadE. Nucleic Acids Res 38:3546–3554. doi:10.1093/nar/gkq097. 20189963 PMC2887963

[55] Richard H, Foster JW 2004 Escherichia coli glutamate- and arginine-dependent acid resistance systems increase internal pH and reverse transmembrane potential. J Bacteriol 186:6032–6041. doi:10.1128/JB.186.18.6032-6041.2004. 15342572 PMC515135

[56] de Jonge R, Takumi K, Ritmeester WS, van Leusden FM 2003 The adaptive response of Escherichia coli O157 in an environment with changing pH. J Appl Microbiol 94:555–560. doi:10.1046/j.1365-2672.2003.01865.x. 12631190

[57] Bergholz TM, Whittam TS 2007 Variation in acid resistance among enterohaemorrhagic Escherichia coli in a simulated gastric environment. J Appl Microbiol 102:352–362. 17241340 10.1111/j.1365-2672.2006.03099.x

[58] Flockhart AF, Tree JJ, Xu X, Karpiyevich M, McAteer SP, Rosenblum R, Shaw DJ, Low CJ, Best A, Gannon V, Laing C, Murphy KC, Leong JM, Schneiders T, La Ragione R, Gally DL 2012 Identification of a novel prophage regulator in Escherichia coli controlling the expression of type III secretion. Mol Microbiol 83:208–223. doi:10.1111/j.1365-2958.2011.07927.x. 22111928 PMC3378721

