# Supplementary material for: Transcriptomic Analysis of Shiga-Toxigenic Bacteriophage Carriage Reveals a Profound Regulatory Effect on Acid Resistance in Escherichia coli
Source: Appl Environ Microbiol. 2015 Oct 30;81(23):8118–25. doi: 10.1128/AEM.02034-15 (PMC4651098; doi:10.1128/AEM.02034-15)
Supplement: Supplemental material [file supp_81_23_8118__index.html]

Transcriptomic Analysis of Shiga-Toxigenic Bacteriophage Carriage Reveals a Profound Regulatory Effect on Acid Resistance in Escherichia coli — Supplemental material 

# Transcriptomic Analysis of Shiga-Toxigenic Bacteriophage Carriage Reveals a Profound Regulatory Effect on Acid Resistance in Escherichia coli

## Supplemental material

- Supplemental file 1 -

  Hierarchical cluster analysis of gene expression in MC1061 and MC1061(ϕ24B) before and after norfloxacin induction (Fig. S1), relative q-RTPCR ratios of *cyoA*, *aceE*, and *mqo* (Fig. S2), bacterial strains, plasmid, and phages used in the study (Table S1), primers used in the study (Table S2), differentially expressed genes (Table S3), and ϕ24B gene expression upon norfloxacin treatment (Table S4).

  PDF, 1.4M
