# Supplementary material for: Transcriptomic Analysis of Shiga-Toxigenic Bacteriophage Carriage Reveals a Profound Regulatory Effect on Acid Resistance in Escherichia coli
Source: Appl Environ Microbiol. 2015 Oct 30;81(23):8118–25. doi: 10.1128/AEM.02034-15 (PMC4651098; doi:10.1128/AEM.02034-15)
Supplement: Supplemental material [file AEM.02034-15_zam999116747so1.pdf]

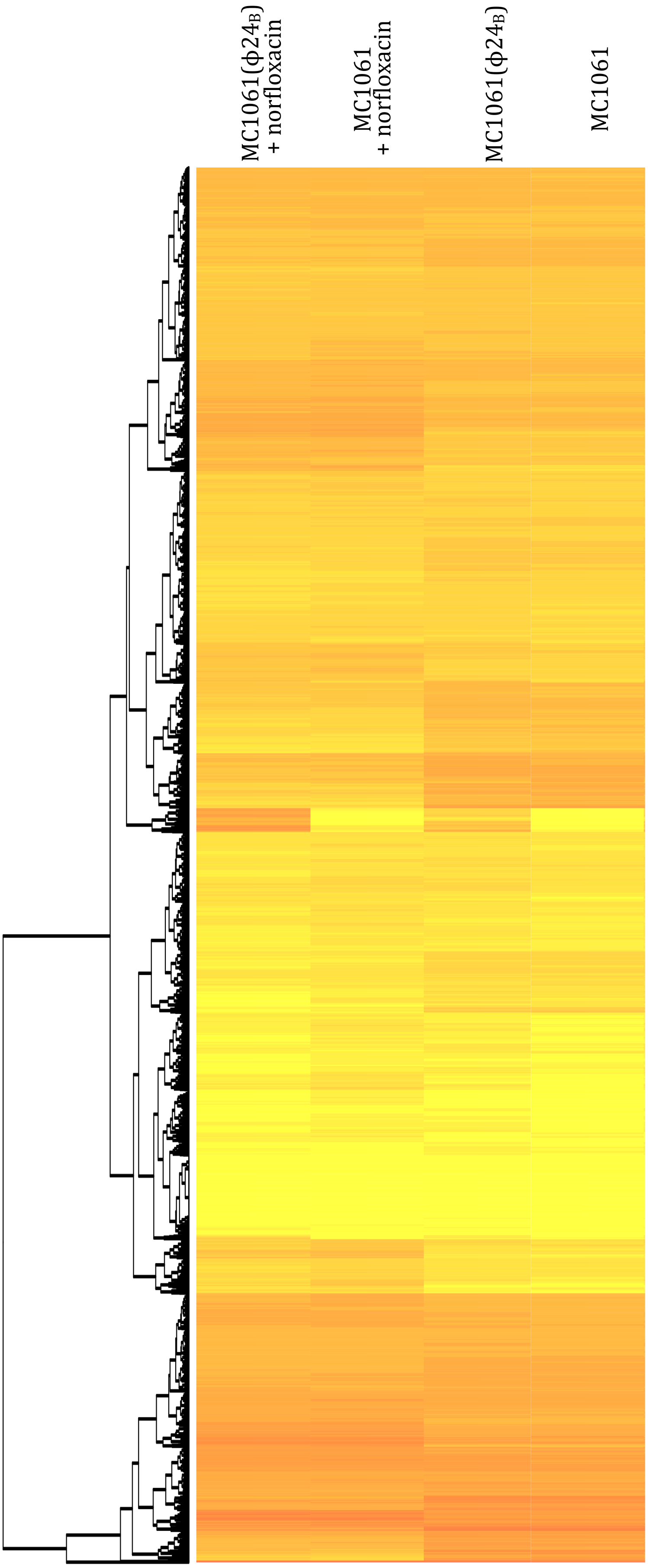

SI Figure 1. Hierarchical cluster analysis of gene expression in MC1061 and MC1061($\phi24_B$) before and after norfloxacin induction. Red and yellow indicate increased and decrease expression respectively.

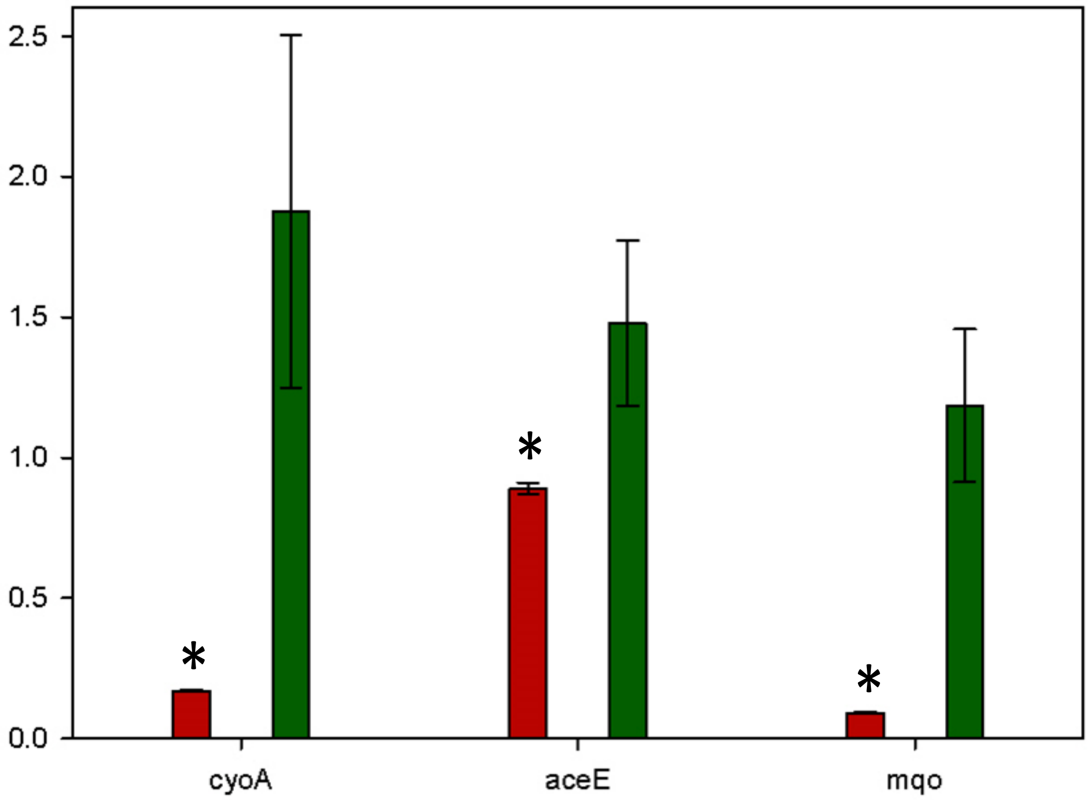

SI Figure 2. Relative q-RTPCR ratios of *cyoA, aceE* and *mqo*. Normalized against the endogenous reference genes *pdxA* and *rraB*. **Red**: MC1061($\phi 24_B$), **green**: MC1061. Error bars represent standard error of the mean (*n*=3). * represents statistically significant values in a two-sample t-test (p value < 0.05)

SI Table 1. Bacterial strains, plasmid and phages used in the study.

E.coli strains, plasmids and phages	Description	Reference
MC1061	*E. coli* K-12 derivative	(1)
$\phi 24_B$::Kan	Stx2-phage, Δ*stxA*$_2$::*aph3*	(1)
TUV93-0	EDL933 with excised Stx1 and Stx2 phages	(2)
pBAD/*Myc*-His C	araBAD, C-myc/His6 epitope, pBR322 ori, AmR	Invitrogen, Paisley, U.K.
p$\phi 24_B$-cI	pBAD carrying $\Phi 24_B$ *cI*	This study
p$\phi 24_B$-cIIcIII	pBAD carrying $\Phi 24_B$ *cII* and *cIII*	This study

SI Table 2. PCR and qPCR primers used in the study.

Oligonucleotide	Sequence (5'-3')	Ref
cI_NcoI	CCATGGTTCAGAATGAAAAAGTG	(3)
cI_SalI	GTCGACGAAGTCACGAACTTTTCAGC	(3)
cII_NcoI	CCATGGAACAAACAAGTTACAGC	This study
cII_SalI	GTCGACTTAAAATTCCATTTGTATT	This study
cII_cIII_phusion	TGGAATTTTAAATGCAATATGCCATTGCAG	This study
cIII_NcoI	CCATGGAATATGCCATTGCAG	This study
cIII_SalI	GTCGACGTCTGGATAGC	This study

Oligonucleotide	Sequence (5'-3')	Primer efficiency*	Ref
gadE_F	GTAGGCAATAAACCCTTCAAGG	99.25	This study
gadE_R	TACTGATGTGATAAGGTGCC		This study
gadX_F	CACAGTTGCTTACTGAGTGTA	99.66	This study
gadX_R	GTGATATCCACAGGATACTG		This study
gadC_F	ACCGGCGTCACTCTGGA	101.03	This study
gadC_R	GTCATTCATCACAATATAGTG		This study
rraB_F	CAGAAGAGCTGGAAGTTGAAG	98.02	This study
rraB_R	TTGTTCAACCTGGGCATCGAT		This study
pdxA_F	GTACGGAAGAGATAGACACCAT	98.19	This study
pdxA_R	GCGTTATCAAGATATTTCGGCTG		This study
mqo_F	CAATACTGATGCTGCCATCG	99.76	This study
mqo_R	CGAACTCTCCTGCGCGACAC		This study
cyoA_F	CGTTGGCGATCAGATGCAG	99.73	This study
cyoA_R	CATCACGGAGTTGGAGGTCAC		This study
aceE_F	GTCTGCAGCACGAAGATGGTC	102.25	This study
aceE_R	GCTGTCATCATGCATGACG		This study
cI_F	GTGAGGGAACGGAGCTACAG	98.54	(4)
cI_R	GCGGCCTTATGCTTTCAATG		(4)
cro_F	AAAGGGCTGTCTATAAGTGG	97.15	(4)
cro_R	GCCACCAGAAATCTCTTCG		(4)
19_F	GGTGCCGGGTGCCTC	98.06	This study
19_R	GGAGGGGCGACTGG		This study
25_F	CGTCTGCTCCACACTGG	98.00	This study
25_R	CGTGACGGAAGATACCACG		This study
28_F	CTGCTCAAAGCTTGTCGC	99.30	This study
28_R	CGGAGCACCCTGAATTCA		This study

35_F	CATCACCTTTCGCCTGTGAT	97.02	This study
35_R	CACTGCGCAATAAGGACAAT		This study

*Primer efficiency calculated applying the formula $E=10^{(-1/slope)}-1$ (5)

SI Table 3. Complete list of bacterial genes differentially expressed in MC1061 and MC1061($\phi 24_B$) upon treatment with norfloxacin.

Gene	Function	Fold change MC1061 ($\phi 24_B$)	P value	Fold change MC1061	P value
**Genes upregulated**					
entE	2,3-dihydroxybenzoate-AMP ligase, enterobactin biosynthesis	110.2	0.001	429.7	0.003
fhuF	Ferric iron reductase Involved in the reduction of ferric iron	101.4	0.001	124.9	0.011
entC	Isochorismate synthase, enterobactin biosynthesis	101.1	0.000	656.6	0.000
efeU	Elemental ferrous iron uptake system	68.5	0.001	58.5	0.014
ybdZ	Putative cytoplasmic protein in enterobactin biosynthesis operon	65.0	0.001	241.0	0.005
entB	Isochorismatase siderophore / Apo-aryl carrier domain, enterobactin biosynthesis	48.9	0.002	116.7	0.006
fes	Enterobactin/Enterochelin esterase, enterobactin biosynthesis	47.4	0.001	128.0	0.004
fecR	Iron(III) dicitrate transmembrane sensor protein	37.8	0.002	31.6	0.038
yjcB	Uncharacterized protein	34.4	0.002	28.2	0.028
entH	Thioesterase, enterobactin biosynthesis	32.6	0.002	63.6	0.012
efeO	Elemental ferrous iron uptake system	28.2	0.003	36.0	0.028
fepA	Outer membrane receptor for ferric enterobactin and colicins B, D	26.9	0.007	89.3	0.046
entS	Enterobactin exporter, iron-regulated	26.7	0.003	80.4	0.009
umuD	Following induction of the SOS response, UmuD is proteolytically processed into UmuD' in a cleavage reaction that depends on activated RecA	25.8	0.005	25.8	0.005
umuC	Error-prone, lesion bypass DNA polymerase V. Binds to processed UmuD protein to form functional DNA pol V (UmuD'2UmuC	24.5	0.005	82.3	0.010
fhuA	Ferrichrome-iron receptor. Acts as a receptor for bacteriophage T5, T1, phi80 and colicin M.	24.1	0.006	104.7	0.007
entF	2,3-dihydro-2,3-dihydroxybenzoate dehydrogenase, enterobactin biosynthesis	23.6	0.011	52.7	0.031
cirA	Colicin I receptor precursor	22.8	0.007	106.4	0.016
recX	Modulates RecA activity through direct physical interaction. May have a regulatory role during the SOS response	21.8	0.009	68.5	0.019

yegD	Putative heat shock protein	20.3	0.012	45.4	0.039
fhuC	Ferric hydroxamate ABC transporter, ATP-binding protein	20.2	0.008	24.1	0.034
entD	4'-phosphopantetheinyl transferase, enterobactin biosynthesis	19.5	0.007	135.4	0.004
entA	2,3-dihydro-2,3-dihydroxybenzoate dehydrogenase, enterobactin biosynthesis	18.5	0.009	65.4	0.013
fepB	ferric enterobactin (enterochelin) binding protein	18.0	0.009	34.4	0.031
fecI	RNA polymerase sigma factor	17.4	0.014	36.9	0.039
fepD	Ferric enterobactin transport system permease	17.2	0.008	47.6	0.021
cspH	Cold shock protein	16.7	0.058	13.0	0.058
fepC	Ferric enterobactin transport ATP-binding protein	16.7	0.009	39.9	0.030
pstS	Phosphate ABC transporter, periplasmic phosphate- binding protein	16.3	0.020	26.5	0.039
nrdF	Ribonucleotide reductase of class Ib (aerobic), beta subunit	16.2	0.009	39.3	0.028
nrdE	Ribonucleotide reductase of class Ib (aerobic), alpha subunit	16.0	0.011	67.1	0.015
fepG	Ferric enterobactin transport system permease	15.7	0.010	145.3	0.007
pdhR	Transcriptional repressor for pyruvate dehydrogenase complex	14.1	0.043	7.3	0.809
rhlE	ATP-dependent RNA helicase	13.7	0.047	9.7	0.277
recA	DNA strand exchange and recombination protein	13.7	0.576	18.7	0.706
recN	DNA repair protein	12.3	0.074	16.5	1.000
Ferredoxin	iron-sulfur proteins that transfer electrons in a wide variety of metabolic reactions	11.8	0.020	25.9	0.025
wcaF	colanic acid biosynthesis acetyltransferase	11.6	0.111	0.5	0.436
cspA	Cold-shock DEAD-box protein A	11.4	0.558	2.5	0.842
nrdI	Electron transport system for the ribonucleotide reductase system	11.2	0.015	67.1	0.006
hycA	Formate hydrogenlyase regulatory protein	10.6	0.021	39.2	0.024
fhuB	Ferric hydroxamate ABC transporter, permease component	10.3	0.024	12.1	0.864
htpX	heat shock membrane protein protein	10.1	0.213	1.1	0.197
phnG	Component of a carbon-phosphorous lyase enzyme	10.0	0.033	31.0	0.020
suhB	Inositol-1-monophosphatase	9.7	0.035	68.5	0.010
carA	Carbamoyl-phosphate synthase small chain	9.5	0.041	18.6	0.684
iscA	Iron binding protein for iron-sulfur cluster assembly	9.4	0.068	4.2	0.775
rnpA	Ribonuclease P protein component	9.2	0.092	5.8	0.467
pspD	Phage shock protein D	9.1	0.043	1.4	0.233
mdtI	spermidine efflux transporter	9.0	0.165	0.1	0.222

nrdH	Electron transport system for the ribonucleotide reductase system	8.9	0.002	65.7	0.018
ansP	L-asparagine permease	8.8	0.046	7.4	0.080
fis	DNA-binding transcriptional dual regulator	8.6	0.079	14.8	0.289
hemP	Hemin uptake protein	8.5	0.047	16.0	0.835
apt	Adenine phosphoribosyltransferase	8.4	0.056	11.6	0.707
stfA	Major fimbrial subunit	8.4	0.085	11.0	0.433
lscS	Cysteine desulfurase	8.2	0.006	6.9	1.000
queD	6-carboxy-5,6,7,8-tetrahydropterin synthase	8.1	0.050	5.2	1.000
rpoA	DNA-directed RNA polymerase alpha subunit	7.9	0.328	4.8	0.916
ycfS	L,D-transpeptidase	7.8	0.065	2.3	0.829
cyoD	Cytochrome O ubiquinol oxidase subunit IV	7.7	0.083	2.1	0.920
cyoA	Cytochrome O ubiquinol oxidase subunit I	7.7	0.084	1.8	0.750
phnD	Phosphonate ABC transporter	7.6	0.038	98.2	0.008
uvrB	Excinuclease ABC subunit B	7.5	0.309	6.6	0.647
plsX	Phosphate:acyl-ACP acyltransferase	7.1	0.110	4.5	1.000
cyoC	Cytochrome O ubiquinol oxidase subunit III	7.1	0.105	1.5	0.750
norR	transcriptional activator	7.1	0.066	2.1	0.414
plsX	Long-chain fatty acid transport protein	7.1	0.090	4.5	0.475
pyrF	Orotidine 5'-phosphate decarboxylase	7.1	0.074	12.3	0.669
acfD	Accessory colonization factor	7.0	0.100	1.9	0.678
	LysR family transcriptional regulator STM2281	7.0	0.040	35.0	0.013
cyoB	Cytochrome O ubiquinol oxidase subunit II	7.0	0.114	1.8	0.207
ibpB	16 kDa heat shock protein B	6.9	0.069	7.3	0.291
phnC	Phosphonate ABC transporter ATP-binding protein	6.8	0.068	78.0	0.904
rnfD	Electron transport complex protein	6.7	0.073	4.0	0.927
yidC	Inner membrane protein translocase	6.6	0.401	4.3	0.349
rph	Ribonuclease PH	6.6	0.087	5.8	0.545
cspI	Cold shock protein	6.6	0.072	4.7	0.895
zraS	Sensor protein of zinc sigma-54-dependent two- component system	6.6	0.082	0.6	0.554
bioB	Biotin synthase	6.5	0.071	4.8	0.973
kdpA	Potassium-transporting ATPase A chain	6.5	0.072	7.9	0.910
dinB	DNA polymerase IV	6.5	0.102	6.5	0.637
fecC	Iron(III) dicitrate transport system permease protein	6.5	0.073	12.0	0.298
wcaB	Colanic acid biosynthesis acetyltransferase	6.4	0.266	8.0	0.976

holD	DNA polymerase III psi subunit	6.4	0.082	6.9	0.559
hscB	Chaperone protein	6.3	0.113	4.8	0.913
mrdA	Penicillin-binding protein 2	6.3	0.120	5.3	0.448
ssb	Single-stranded DNA-binding protein	6.3	0.167	2.1	0.948
ligT	2'-5' RNA ligase	6.3	0.074	2.7	0.500
kdpB	Potassium-transporting ATPase B chain	6.2	0.082	2.8	0.836
typA	GTP-binding protein TypA/BipA	6.1	0.329	2.7	0.604
cspB	Cold shock protein	6.0	0.099	23.4	0.206
piuC	Iron-uptake factor	6.0	0.086	17.3	0.516
cmk	Cytidylate kinase	6.0	0.130	8.2	0.753
iscU	Iron-sulfur cluster assembly scaffold protein	5.9	0.128	7.5	0.715
betT	High-affinity choline uptake protein	5.9	0.119	5.5	0.764
betI	HTH-type transcriptional regulator	5.9	0.125	3.3	0.889
zraR	Response protein of zinc sigma-54-dependent two- component system	5.8	0.085	1.0	0.826
fusA	Translation elongation factor G	5.8	0.252	3.4	0.605
phnC	Phosphonate ABC transporter	5.3	0.065	78.0	0.012
piuC	Iron-uptake factor	4.6	0.086	17.4	0.047
yoaA	DinG family ATP-dependent helicase	4.4	0.125	5.5	0.989
cynT	Carbonic anhydrase	4.3	0.132	6.2	0.696
cspF	Cold shock protein	4.3	0.092	20.8	0.412
betA	Choline dehydrogenase	4.3	0.135	2.9	0.586
aceF	Dihydrolipoamide dehydrogenase	4.3	0.368	3.6	0.822
nth	Endonuclease III	4.2	0.093	4.1	0.870
glpE	Thiosulfate sulfurtransferase	4.2	0.097	2.7	0.592
rnfE	Electron transport complex protein	4.2	0.096	2.9	0.295
kdpF	Potassium-transporting ATPase C chain	4.2	0.097	3.5	0.726
hscA	Chaperone protein	4.1	0.144	2.9	0.627
cyoE	Heme O synthase, protoheme IX farnesyltransferase	4.1	0.139	1.8	0.127
dgoT	D-galactonate transporter	4.1	0.114	4.4	0.366
betB	Betaine aldehyde dehydrogenase	4.0	0.146	3.4	0.891
slp	Starvation lipoprotein Slp paralog	4.0	0.115	4.2	0.998
	Protein Implicated in DNA repair function with RecA and MutS	4.0	0.117	7.1	0.685
phoH	Phosphate starvation-inducible protein	3.9	0.107	3.1	0.063
pstB	Phosphate transport ATP-binding protein	3.9	0.129	5.2	0.837

ruvC	Crossover junction endodeoxyribonuclease	3.9	0.127	3.0	0.896
sixA	Phosphohistidine phosphatase	3.8	0.134	3.1	0.508
speE	Spermidine synthase	3.8	0.148	3.7	0.584
fecB	Iron(III) dicitrate transport system	3.7	0.120	19.6	0.047
hycC	Formate hydrogen lyase	3.7	0.117	23.4	0.041
hycB	Formate hydrogen lyase	3.7	0.136	31.1	0.039
rnfG	Electron transport complex protein	3.5	0.095	2.4	0.411
	proteinase inhibitor	3.2	0.686	42.0	0.028
hycD	Formate hydrogen lyase	3.0	0.168	16.3	0.049
phnH	Component of a carbon-phosphorous lyase enzyme	2.5	0.237	78.0	0.003
phnF	Transcriptional regulator of phosphonate uptake	2.3	0.305	92.0	0.009
ygeP	Uncharacterized protein	2.1	0.281	28.4	0.045
phnE	Phosphonate ABC transporter	2.0	0.369	115.0	0.017
phnI	Component of a carbon-phosphorous lyase enzyme	1.8	0.358	82.5	0.011
phnK	Component of a carbon-phosphorous lyase enzyme	1.5	0.494	42.5	0.019
**Genes downregulated**					
gutA	PTS system, glucitol/sorbitol-specific IIA component	202.5	0.000	169.5	0.000
malE	Maltose/maltodextrin ABC transporter, substrate binding periplasmic protein	162.7	0.975	413.4	0.974
gutD	Sorbitol-6-phosphate 2-dehydrogenase	157.3	0.988	138.5	0.004
malK	Maltose/maltodextrin transport ATP-binding protein	143.9	0.100	251.2	0.001
srlE	PTS system, glucitol/sorbitol-specific IIB component	138.9	0.990	197.9	0.002
lamB	Maltoporin	115.3	0.982	254.5	0.956
treB	PTS system, trehalose-specific IIB component	113.8	0.934	147.6	0.944
treC	Trehalose-6-phosphate hydrolase	111.2	0.972	174.1	0.966
ompW	Outer membrane protein W precurso	109.4	0.986	66.6	0.007
fliA	RNA polymerase sigma factor for flagellar operon	104.0	0.005	141.0	0.002
fliL	Flagellar biosynthesis protein	99.0	0.005	28.4	0.010
tnaA	Tryptophanase	98.6	0.837	658.6	0.919
malF	Maltose/maltodextrin ABC transporter, permease protein	94.0	0.037	91.8	0.004
gatC	PTS system, galactitol-specific IIB component	92.0	0.004	99.4	0.001
ygeV	Uncharacterized sigma-54-dependent transcriptional regulator	86.2	0.005	48.0	0.004
	Pyridine nucleotide-disulphide oxidoreductase family	83.1	0.005	52.2	0.004

ansA	L-asparaginase	77.3	0.959	60.1	0.676
malM	Maltose operon periplasmic protein	76.5	0.048	71.2	0.004
tnaB	tryptophan permease	75.5	0.314	670.0	0.955
cstA	Carbon starvation protein A paralog	73.0	0.981	77.6	0.274
glpT	Glycerol-3-phosphate transporte	66.1	0.779	80.8	0.010
gatD	Galactitol-1-phosphate 5-dehydrogenase	62.6	0.010	86.3	0.010
hybA	Hydrogenase-2 operon protein	62.3	0.032	55.9	0.015
malG	Maltose/maltodextrin ABC transporter, permease protein	59.8	0.030	46.5	0.018
manX	PTS system, mannose-specific IIAB component	59.1	0.915	42.6	0.020
psuG	Pseudouridine 5'-phosphate glycosidase	58.5	0.009	7.9	0.111
glpQ	Glycerophosphoryl diester phosphodiesterase	57.8	0.967	26.6	0.033
preA	Dihydropyrimidine dehydrogenase	57.0	0.010	65.6	0.013
cadB	Lysine/cadaverine antiporter membrane protein	55.3	0.008	14.1	0.062
potD	periplasmic spermidine putrescine-binding protein	54.8	0.018	20.3	0.043
tdcD	propionate/acetate kinase	52.5	0.010	73.6	0.011
GALNS	N-acetylgalactosamine 6-sulfate sulfatase	51.4	0.011	31.9	0.027
gudP	D-galactarate permease	51.3	0.010	27.2	0.011
aphA	periplasmic phosphatase/phosphotransferase	47.5	0.011	26.3	0.033
gadC	glutamate/gamma-aminobutyrate antiporter	46.9	0.938	7.3	0.111
dmsA	Anaerobic dimethyl sulfoxide reductase chain A	46.7	0.239	64.2	0.013
tdcC	L-threonine transporter	45.1	0.037	21.2	0.041
manY	PTS system, mannose-specific IIC component	44.3	0.926	64.2	0.013
garL	2-dehydro-3-deoxyglucarate aldolase	44.0	0.013	32.7	0.026
dmsB	Anaerobic dimethyl sulfoxide reductase chain B	43.6	0.044	74.7	0.011
mglB	D-galactose-binding periplasmic protein	43.1	0.053	46.6	0.018
nanA	N-acetylneuraminate lyase	42.9	0.117	25.6	0.034
ykgE	L-lactate dehydrogenase, Fe-S oxidoreductase subunit	41.8	0.013	57.8	0.015
tdcB	Threonine dehydratase	41.3	0.027	25.4	0.034
lsrB	Autoinducer 2 (AI-2) ABC transport system, periplasmic AI-2 binding protein	40.7	0.013	23.4	0.037
xylF	Xylose ABC transporter periplasmic protein	40.5	0.015	11.1	0.079
gatC	PTS system, galactitol-specific IIA component	39.7	0.028	28.9	0.010
potC	Spermidine Putrescine ABC transporter permease	38.0	0.018	18.4	0.047
yneH	Glutaminase	36.9	0.083	38.5	0.022
fadH	2,4-dienoyl-CoA reductase [NADPH]	36.8	0.018	2.1	0.415

galT	Galactose-1-phosphate uridylyltransferase	36.3	0.843	19.0	0.046
ybaT	Inner membrane transport protein	34.7	0.090	24.7	0.035
hybB	Ni/Fe-hydrogenase 2 B-type cytochrome subunit	33.8	0.086	34.8	0.025
galK	Galactokinase	33.7	0.164	30.6	0.028
caiF	Transcriptional activatory protein CaiF	32.8	0.000	37.2	0.008
ldcC	Lysine decarboxylase	32.1	0.028	11.7	0.075
flgB	Flagellar basal-body rod protein	31.8	0.019	43.6	0.020
nanT	Sialic acid transporter	30.9	0.064	8.0	0.109
cspD	Cold shock protein	30.9	0.250	38.1	0.023
lsrF	Autoinducer 2 (AI-2) aldolase	30.8	0.021	20.6	0.042
uspF	Universal stress protein F	29.8	0.042	16.4	0.053
patD	4-aminobutyraldehyde dehydrogenase	29.4	0.051	22.8	0.038
aldB	Aldehyde dehydrogenase B	28.6	0.102	29.5	0.029
gatZ	Tagatose-6-phosphate kinase	27.8	0.886	25.8	0.034
	DUF1440 domain-containing membrane protein	27.7	0.028	23.4	0.037
potA	Putrescine transport ATP-binding protein	27.6	0.029	37.5	0.023
uspD	Universal stress protein D	26.6	0.027	16.5	0.053
idnD	L-idonate 5-dehydrogenase	26.5	0.033	13.1	0.028
garD	D-galactarate dehydratase	26.3	0.047	25.4	0.034
ftnB	Ferritin-like protein	25.5	0.041	35.3	0.024
flgE	Flagellar hook protein	25.0	0.051	46.9	0.006
tdcF	Hypothetical protein TdcF in cluster with anaerobic degradation of L-threonine to propionate	24.8	0.029	26.1	0.033
xynT	Xyloside transporter	24.8	0.033	22.3	0.013
	Putative fimbrial-like protein	24.5	0.039	7.5	0.065
fliM	Flagellar motor switch protein	24.3	0.030	15.1	0.030
dmsC	Anaerobic dimethyl sulfoxide reductase chain C	23.9	0.061	37.4	0.007
yagH	Beta-xylosidase	23.0	0.042	17.3	0.020
tdcA	Threonine catabolic operon transcriptional activator	22.8	0.043	4.8	0.144
adh	Alcohol dehydrogenase	22.3	0.547	20.8	0.605
fndI	Formate dehydrogenase N gamma subunit	22.3	0.046	36.0	0.006
mglA	Galactose/methyl galactoside ABC transport system, ATP-binding protein	22.0	0.048	8.5	0.064
yihP	Glucuronide transport protein	22.0	0.074	0.5	0.796
gutM	Glucitol operon activator protein	21.8	0.041	49.2	0.003

rbsB	Ribose ABC transport system, periplasmic ribose- binding protein	21.7	0.627	28.1	0.745
gne	UDP-N-acetylglucosamine 4-epimerase	21.5	0.747	15.9	0.692
uxaC	Uronate isomerase	21.4	0.127	7.5	0.091
malT	Transcriptional activator of maltose regulon	21.4	0.563	18.2	0.036
fdoH	Formate dehydrogenase O beta subunit	21.3	0.059	53.5	0.005
agp	Glucose-1-phosphatase	21.2	0.546	18.7	0.033
xanP	Xanthine permease	21.0	0.081	13.2	0.032
gadB	Glutamate decarboxylase	20.9	0.056	4.2	0.172
pepE	Alpha-aspartyl dipeptidase Peptidase E	20.8	0.051	31.3	0.008
gutQ	D-arabinose 5-phosphate isomerases	20.7	0.067	12.3	0.038
agaZ	Tagatose-6-phosphate kinase	20.5	0.040	2.9	0.245
srlR	Glucitol operon repressor	20.2	0.062	15.1	0.026
talA	Transaldolase	20.2	0.039	6.8	0.086
fndG	Formate dehydrogenase N alpha subunit	19.6	0.085	100.9	0.004
gatY	Tagatose-1,6-bisphosphate aldolase	19.3	0.745	19.9	0.732
tdcE	Pyruvate formate-lyase	19.3	0.060	32.7	0.012
sdhB	Succinate dehydrogenase iron-sulfur protein	19.1	0.881	19.6	0.753
dcuC	C4-dicarboxylate transporter	19.1	0.051	15.8	0.024
gcvT	Glycine cleavage system H protein	19.0	0.831	16.6	0.041
yihT	Aldolase	19.0	0.048	1.5	0.524
fucO	Lactaldehyde reductase	18.9	0.052	14.3	0.029
IUNH	Inosine-uridine preferring nucleoside hydrolase	18.0	0.219	7.8	0.087
fliS	Flagellar biosynthesis protein	18.0	0.108	4.6	0.189
molR	Molybdate metabolism regulator	18.0	0.108	0.7	0.959
yfaW	Uncharacterized protein	17.7	0.048	12.8	0.036
fliN	Flagellar motor switch protein	17.2	0.056	47.5	0.005
glpK	Glycerol kinase	16.9	0.920	26.8	0.814
nanE	N-acetylmannosamine-6-phosphate 2-epimerase	16.9	0.069	6.6	0.092
fliF	Flagellar M-ring protein	16.8	0.053	15.1	0.029
lsrG	Autoinducer 2 (AI-2) modifying protein	16.6	0.061	8.6	0.062
araE	Arabinose-proton symporter	16.2	0.878	5.6	0.112
xylG	D-xylose transport ATP-binding protein	16.2	0.055	5.4	0.108
nrfA	Cytochrome c552 precursor	16.2	0.080	24.1	0.014
aldA	Glycolaldehyde dehydrogenase	15.9	0.153	41.9	0.010
rbsK	Ribokinase	15.9	0.890	13.0	0.051

ydcE	Probable tautomerase	15.9	0.057	3.4	0.210
hdeA	chaperone	15.6	1.000	2.7	0.325
rspA	Starvation sensing protein	15.5	0.077	3.6	0.239
flgF	Flagellar basal-body rod protein	15.5	0.064	25.6	0.013
dmsB	Anaerobic dimethyl sulfoxide reductase chain B	15.3	0.081	47.1	0.005
csiE	Stationary phase inducible protein	15.2	0.323	9.2	0.070
rbsC	Ribose ABC transport system, permease protein	15.1	0.865	11.9	0.624
gldA	Glycerol dehydrogenase	15.1	0.134	14.6	0.033
ygeW	Aspartate/ornithine carbamoyltransferase family protein protein	15.1	0.063	91.6	0.002
uxaA	Altronate hydrolase	15.0	0.138	7.6	0.082
hdeD	Membrane transporter	14.8	0.154	1.4	0.605
dapA	Dihydrodipicolinate synthase	14.8	0.846	16.1	0.747
yegT	Putative nucleoside transporter	14.8	0.067	7.4	0.077
pstI	Phosphoenolpyruvate-protein phosphotransferase of PTS system	14.8	0.065	2.9	0.274
zraP	Zinc resistance-associated protein	14.6	0.218	5.0	0.127
rhaR	L-rhamnose operon transcriptional activator	14.6	0.063	1.0	0.806
uidB	Glucuronide transporter	14.6	0.064	6.6	0.088
gntP	Fructuronate transporter	14.6	0.074	7.2	0.086
ykgF	Predicted L-lactate dehydrogenase, Iron-sulfur cluster-binding subunit	14.4	0.090	25.9	0.015
yhjA	Cytochrome c551 peroxidase	14.4	0.072	8.1	0.073
fliO	Flagellar biosynthesis protein	13.8	0.076	18.0	0.017
hoxL	Uptake hydrogenase large subunit	13.8	0.087	17.4	0.023
melB	Melibiose carrier protein, Na+/melibiose symporter	13.8	0.073	5.9	0.110
dctA	Aerobic C4-dicarboxylate transporter for fumarate, L-malate, D-malate, succunate	13.7	0.200	4.7	0.166
hdeB	Chaperone	13.5	0.206	2.2	0.385
atoC	Acetoacetate metabolism regulatory protein	13.5	0.076	13.6	0.035
murQ	N-acetylmuramic acid 6-phosphate etherase	13.4	0.081	13.4	0.034
yfaX	Uncharacterized transcriptional regulator	13.3	0.080	1.5	0.529
hybF	[NiFe] hydrogenase nickel incorporation protein	13.3	0.081	4.9	0.142
	Transcriptional regulator, IclR family	13.3	0.080	2.1	0.400
gadA	Glutamate decarboxylase	13.2	0.084	2.4	0.339
ucpA	Oxidoreductase	13.2	0.582	9.2	0.080
gcvP	Glycine dehydrogenase	13.1	0.887	22.3	0.791
atoS	Sensory histidine kinase	13.1	0.080	12.4	0.040

cusC	Cation efflux system protein	13.1	0.197	0.4	0.631
gadW	HTH-type transcriptional regulator	13.0	0.083	1.6	0.538
frdA	Fumarate reductase subunit C	12.9	0.304	13.5	0.042
dppA	Dipeptide-binding ABC transporter, periplasmic substrate-binding component	12.9	0.268	9.8	0.061
malP	Maltodextrin phosphorylase	12.9	0.998	11.3	0.063
acrB	Membrane fusion protein of RND family multidrug efflux pum	12.9	0.105	4.1	0.180
cydA	Cytochrome d ubiquinol oxidase subunit I	12.8	0.778	20.2	0.786
lsrB	Autoinducer 2 (AI-2) ABC transport system, fused AI2 transporter subunits and ATP-binding component	12.8	0.079	5.5	0.101
melA	Alpha-galactosidase	12.8	0.095	8.6	0.061
uspG	Universal stress protein G	12.6	0.171	15.4	0.026
sdaC	Serine transporter	12.6	0.123	12.8	0.035
yphF	Predicted sugar ABC transport system, periplasmic binding protein	12.6	0.088	5.5	0.133
eutQ	Ethanolamine utilization protein	12.5	0.116	3.4	0.205
nikC	Nickel transport system permease protein	12.4	0.085	9.0	0.058
uxaB	Altronate oxidoreductase	12.3	0.107	5.8	0.108
tdcG	Predicted L-serine dehydratase	12.2	0.087	13.9	0.030
fliQ	Flagellar biosynthesis protein	12.0	0.168	5.5	0.133
wrbA	Flavoprotein	11.8	0.878	14.2	0.053
acs	Acetyl-coenzyme A synthetase	11.8	0.106	20.0	0.023

SI Table 4. $\phi 24_B$ gene expression upon norfloxacin treatment.

Gene	Function	Fold change	Pvalue
vb_24B+1c	Unknown	72.34	0.007
vb_24B+2c	Unknown	73.34	0.009
vb_24B+4c	Unknown	105.44	0.004
vb_24B+5c	Unknown	99.73	0.001
vb_24B+7c	Unknown	72.53	0.007
vb_24B+8c	Unknown	111.42	0.003
vb_24B+9c	Unknown	119.5	0.001
vb_24B+14	Unknown	103.38	0.001
vb_24B+15	Unknown	94.57	0.001
vb_24B+16	Unknown	141	0.000
vb_24B+17	Unknown	36.35	0.002
vb_24B+23	Unknown	38.73	0.009
vb_24B+26	Unknown	90.4	0.001
vb_24B+31	Unknown	28.12	0.011
vb_24B+33	adaptive-response sensory kinase	51.93	0.008
vb_24B+34	Unknown	51.01	0.011
vb_24B+35	Unknown	51.94	0.005
vb_24B+36	Unknown	21.75	0.023
vb_24B+39	Unknown	22.32	0.021
vb_24B+40	Unknown	15.5	0.023
vb_24B+41	Unknown	15.82	0.010
vb_24B+41	Unknown	15.72	0.011
vb_24B+44	Unknown	8.34	0.039
vb_24B+45	Unknown	7.01	0.047
vb_24B+52c	Unknown	10.5	0.023
ninA	Uncharacterized phage-encoded protein	71.33	0.000
ninB	putative DNA N-6-adenosine methyltransferase	68.94	0.009
ninG	putative recombination endonuclease	124.02	0.003
ninH	Uncharacterized phage-encoded protein	89.9	0.000
ninE	Uncharacterized phage-encoded protein	143.07	0.000
S	Host cell lysis	133.64	0.002
R	Associated with endolysin/autolysin	118	0.003
Q	Antiterminator	50.93	0.005
stx	Shiga toxin	95.77	0.000
cII	Transcriptional activator	47.45	0.002
cIII	Regulatory protein	70.35	0.002
cro	Transcriptional activator/repressor	44.9	0.008
d_ant		95.60	0.004
ant	Uncharacterized protein	27.32	0.018
roi	Uncharacterized protein	88.55	0.003
Rz	Host cell lysis	49.73	0.005
Rz1	Host cell lysis	86.33	0.000
kil	Host killing; lambda Kil superfamily	83.63	0.003
hyp+c4-zinc+finger	Unknown	76.01	0.006
Sterm	Terminase, small subunit	74.9	0.004
lar	putative restriction alleviation protein	63.84	0.004
hyp+33	Unknown	49.84	0.005
xis	excisionase	51.16	0.002
hyp+DAM	Unknown	101.17	0.004
vb_24B+3c	Unknown	76.35	0.272

vb_24B+10	Unknown	0.6	0.272
vb_24B+11c	Unknown	43.17	0.273
vb_24B+12c	Unknown	3.63	0.141
vb_24B+19c	Unknown	1.63	0.400
vb_24B+20c	Unknown	3.44	0.136
vb_24B+24	Unknown	47.3	0.626
vb_24B+27c	Unknown	1	1
vb_24B+28	Unknown	49.01	0.837
vb_24B+29	Unknown	31.71	0.623
vb_24B+30c	Unknown	0.81	0.848
vb_24B+32c	Unknown	1	0.797
Lterm	Terminase, large subunit	39.17	0.132
vb_24B+37c	Unknown	1.63	0.400
vb_24B+47	Unknown	7.30	0.059
vb_24B+48	Unknown	5.63	0.309
vb_24B+51c	Unknown	0.71	0.879
Int	Integrase	1.73	0.271
vb_24B+6c	Unknown	87.75	0.272
res	putative holliday junction resolvase	1.16	0.532
vb_24B+13c	Unknown	0.65	0.908
exo	Exonuclease	98.02	0.273
bet	possesses large RecT domain	89.25	0.273
gam	Host nuclease inhibitor protein	136.73	0.273
ssb	putative single-stranded DNA binding protein	78.65	0.273
stk	Serine/Threonine protein kinases	1.81	0.329
bor	lambda *bor* increases the resistance of *E. coli* to serum	1.43	0.443
cI	repressor protein	1.23	0.446
lom	Outer membrane protein beta-barrel domain	3.00	0.140
port	putative portal protein	29.89	0.526
O	DNA replication protein	64.99	0.606
N	anti-terminator	47.46	0.736
vb_24B+21	Unknown	23.99	0.745
P	DNA replication protein	42.22	0.804

Referecnes:

1. **James CE**, **Stanley KN**, **Allison HE**, **Flint HJ**, **Stewart CS**, **Sharp RJ**, **Saunders JR**, **McCarthy AJ**. 2001. Lytic and lysogenic infection of diverse *Escherichia coli* and *Shigella* strains with a verocytotoxigenic bacteriophage. Appl. Environ. Microbiol. **67**:4335–4337.
2. **Campellone KG**, **Roe AJ**, **Løbner-Olesen A**, **Murphy KC**, **Magoun L**, **Brady MJ**, **Donohue-Rolfe A**, **Tzipori S**, **Gally DL**, **Leong JM**, **Marinus MG**. 2007. Increased adherence and actin pedestal formation by dam-deficient enterohaemorrhagic *Escherichia coli* O157:H7. Molecular Microbiology **63**:1468–1481.
3. **Fogg PCM**, **Rigden DJ**, **Saunders JR**, **McCarthy AJ**, **Allison HE**. 2011. Characterization of the relationship between integrase, excisionase and antirepressor activities associated with a superinfecting Shiga toxin encoding bacteriophage. Nucleic Acids Res **39**:2116–2129.
4. **Riley LM**, **Veses-Garcia M**, **Hillman JD**, **Handfield M**, **McCarthy AJ**, **Allison HE**. 2012. Identification of genes expressed in cultures of *E. coli* lysogens carrying the Shiga toxin-encoding prophage $\Phi 24_B$. BMC Microbiol **12**:42.
5. **Rasmussen R**. 2012. Quantification of the LightCycler, pp. 21–34. *In* Meuer, S, Wittwer, C, Nakagawara, K (eds.), Rapid cycle real-time PCR, methods and applications. Springer Science & Business Media, Berlin Heidelberg.
